# Trisubstituted 4f- and 4d tungstoantimonates as artificial phosphoesterases for nerve agent degradation†

**DOI:** 10.1039/d2cc02223k

**Published:** 2022-06-13

**Authors:** Elias Tanuhadi, Annette Rompel

**Affiliations:** Universität Wien, Fakultät für Chemie, Institut für Biophysikalische Chemie 1090 Wien Austria annette.rompel@univie.ac.at https://www.bpc.univie.ac.at

## Abstract

Three new trisubstituted 4f- and 4d tungstoantimonates (TA) K_3_Na_21_[(M(CH_3_COO))_3_(HPO_3_)(WO_4_)(SbW_9_O_33_)_3_]·*n*H_2_O {M_3_(HPO_3_)Sb_3_W_28_} (M = Gd^III^, Y^III^, Yb^III^, *n* = 35–36) were synthesized using a double-template synthetic approach. Following their characterization in the solid state employing single- and powder X-ray diffraction (XRD), IR-spectroscopy, and elemental – and thermogravimetric analyses (TGA), {M_3_(HPO_3_)Sb_3_W_28_} were subjected to a comprehensive set of solution characterization methods including UV/vis- and multinuclear ^31^P and ^13^C NMR spectroscopy. All representatives were shown to be highly active, recyclable, and stable Lewis-acid catalysts towards the nerve agent simulant *O*,*O*-dimethyl *O*-(4-nitrophenyl) phosphate (DMNP) at neutral pH (in Tris–HCl [125 mM] at pD 7.0 25 °C). Control experiments showing catalytic activity of the unsubstituted trilacunary **TA** [SbW_9_O_33_]^9−^ suggest the non-innocence of Tris in the DMNP hydrolysis for the first time.

## Abstract 2

With 3 Mio. people worldwide being yearly exposed to organophosphates (OPs), accounting for approximately 300 000 deaths, OPs are a current threat to mankind. This work reports on {M_3_(HPO_3_)Sb_3_W_28_} and **{SbW_9_}** as recyclable OP degradation catalysts.

## Graphical abstract

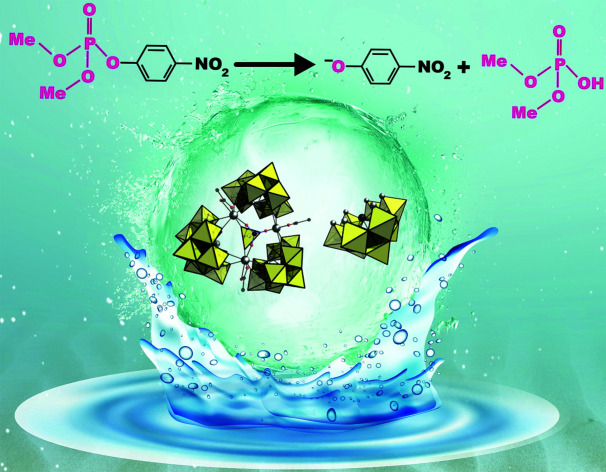

Polyoxometalates (POMs)^1^ represent a broad class of anionic inorganic clusters with versatile structural topologies resulting in a variety of chemical and physical properties which can be modulated by molecular design. These features make them attractive materials in a wide range of fields like catalysis,^2^ supramolecular chemistry,^3^ magneto chemistry,^4^ biological chemistry^5^ including protein crystallography^6^ and subject of challenging interdisciplinary research.^7^ Their inherently high negative charge, water solubility and modular properties render POMs promising candidates for catalytic studies.^8^

Among the various targets of POM based catalysis, phosphodiester bonds, which are characterized by a high stability with a half-life of 130 000 years towards hydrolysis under physiological conditions (at neutral pH and 25 °C),^9^ have been the focus of research as their controlled hydrolysis under ambient turnover conditions represents a significant goal for *e.g.*, biotechnological applications.^10^ A timely example for the applied hydrolysis of phosphodiester bonds is the removal of toxic organophosphorus (OP) esters containing P–X bonds (X = CN, SR)^11,12^ that represent a significant threat to the environment due to their toxicity by inactivating acetylcholinesterase leading to even death in high doses.^13,14^ Previous work demonstrated lanthanide(iii)- and zirconium(iv) ions as highly suitable active sites in the design of artificial phosphoesterases considering their high charge density and coordination number, as well as fast ligand exchange rates.^15^ In this context, the heterogeneous nature of conventionally used zirconium(iv) incorporating hydrolytic catalysts based on metal oxides, metal hydroxides, and metal–organic frameworks (MOFs)^15^ renders the active sites’ exact structure widely elusive. Hence, driven by molecular catalysts featuring well-defined Lewis-acid active sites and a known speciation under turnover conditions, thereby contributing to understanding the hydrolytic mechanism and crucial factors governing the decontamination process, previous efforts have been made by Hill and co-workers thoroughly investigating the Zr-substituted POM, {[*α*-PW_11_O_39_Zr(*μ*-OH)(H_2_O)]_2_}^8−^ as a homogeneous catalyst for the hydrolysis of nerve agent simulants under buffered conditions.^16^ While these studies led to significant mechanistic insights on POMs’ phosphoesterase activity, the applied Zr-POM dimer is unstable and dissociates into its monomeric form under turnover conditions. Consequently, the synthesis of a stable, recyclable homogeneous Lewis-acid POM catalyst with a known speciation chemistry under turnover conditions represents the next step in the development of nerve-agent degradation systems.

Herein, we employ a double-template synthetic approach for the targeted design of a series of three new trisubstituted tungstoantimonates with the general sum formula K_3_Na_21_[(M(CH_3_COO))_3_(HPO_3_)(WO_4_)(SbW_9_O_33_)_3_]·*n*H_2_O {M_3_(HPO_3_)Sb_3_W_28_} (M = Gd^III^, Y^III^, Yb^III^, *n* = 35–36). Owing to their pronounced water solubility (∼8 mM) and high number of incorporated accessible Lewis-acid metal centers, {M_3_(HPO_3_)Sb_3_W_28_} are ideal candidates as water-stable, recyclable homogeneous artificial phosphoesterases for the Lewis-acidic degradation of the nerve agent simulant *O*,*O*-dimethyl *O*-(4-nitrophenyl) phosphate (DMNP) (Scheme S1, ESI†) under ambient reaction conditions (25 °C, pD = 7.0). The embedment of the {HPO_3_} motif in the isostructural series {M_3_(HPO_3_)Sb_3_W_28_} allowed ^31^P NMR experiments on reaction mixtures offering insights into the catalysts’ solution behavior in the presence of the DMNP substrate and aiding in the disentanglement of the elsewise mostly elusive speciation of homogeneous POM-catalysts under catalytic turnover conditions.

{M_3_(HPO_3_)Sb_3_W_28_} were prepared employing a double-template synthetic approach^17^ starting from the literature known trilacunary tungstoantimonate precursor Na_9_[*B*-SbW_9_O_33_]·19.5 H_2_O {SbW_9_}^18^ that was chosen based on its previously reported affinity towards 3d- and 4f electrophiles^19^ and the presence of a lone-pair preventing *in situ* formation of a fully closed Keggin sphere therewith allowing the generation of more complex POM-architectures.^20^ To a stirred suspension of {SbW_9_}, Na_2_WO_4_ and KCl in a 2 : 1 mixture of sodium acetate buffer ([2 M], pH = 5.5) and water, phosphorous acid and the corresponding Lewis-acid metal salt were added followed by stirring at 25 °C for 30 min and subsequent heating at 90 °C for an hour (Scheme 1). Slow evaporation of the solution in an uncovered beaker at 20 °C led to formation of a yellow oil within 24 h (Fig. S5A, ESI†) and eventually afforded yellow block shaped single crystals crystallizing from the oil after covering the beaker for another two days (Fig. S5B, ESI†). Note that higher or lower acetate buffer concentrations led to immediate formation of yellow precipitates or no crystals, respectively.

**Scheme 1:**
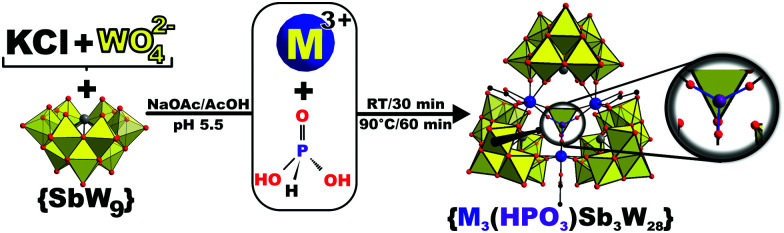
Schematic representation showing the double-templated synthesis of {M_3_(HPO_3_)Sb_3_W_28_} (M = Gd^III^, Y^III^, Yb^III^) starting from a buffered solution containing KCl, the trilacunary {SbW_9_} precursor and the first template added as Na_2_WO_4_. Addition of the corresponding metal center and phosphorous acid acting as the second template accompanied by room temperature stirring and heating lead to formation of the target compound featuring a labile acetate ligand bound to each of the three 8-fold coordinated Lewis-acid metal centers with square-antiprismatic symmetry. Blue and red spheres represent the M- and oxygen ions, respectively. Grey for Sb^III^, purple for P^V^ and yellow polyhedra for {WO_6_}.

Single crystal X-ray diffraction (SXRD) measurements were performed (Table S5, ESI†) on {Gd_3_(HPO_3_)Sb_3_W_28_} (Tables S6 and S7, ESI†), {Y_3_(HPO_3_)Sb_3_W_28_} (Tables S8 and S9, ESI†), and {Yb_3_(HPO_3_)Sb_3_W_28_} (Tables S10 and S11, ESI†) revealing that all isostructural representatives contain 4d- or 4f metal centers that are each coordinated by an acetate-ligand and the central {HPO_3_} group, respectively (Scheme 1 and Fig. S10, ESI†). Subsequent solid – and solution state characterization featuring elemental analyses, IR spectroscopy (Fig. S1 and Table S1, ESI†), thermogravimetric analyses (TGA; Fig. S2–S4 and Tables S2–S4, ESI†), powder XRD (PXRD; Fig. S7, ESI†), and UV/vis spectroscopy (Fig. S8, ESI†) reinforce the compounds’ elemental composition and homogeneity.

Following ^13^C – and ^31^P NMR studies on the diamagnetic representative {Y_3_(HPO_3_)Sb_3_W_28_} of the isostructural series demonstrating {M_3_(HPO_3_)Sb_3_W_28_} to exhibit pre-catalytic solution stability (Fig. S11A, B, S12 and Tables S12, S13, ESI†) and showcasing the three acetate ligands to be easily exchangeable (Fig. S9 and S10A, B, ESI†) which is a prerequisite for hydrolytic activity, {M_3_(HPO_3_)Sb_3_W_28_} were subjected to catalytic experiments employing *O*,*O*-dimethyl *O*-(4-nitrophenyl) phosphate (DMNP), a commonly used model substrate for degradation studies, at room temperature (25 °C) and physiological pH (125 mM Tris–HCl, pD 7).^21^ The stepwise hydrolysis of DMNP to nitrophenol and the side product dimethyl phosphate (DMP) was monitored employing ^31^P NMR spectroscopy on aliquots taken after 0, 269, 1362, 1735, 2776, 3178, and 4079 min of reaction (Scheme S1 and Fig. 1, Fig. S13, S14, ESI†). A stepwise disappearance of the singlet corresponding to the substrate DMNP, and gradual appearance of a singlet attributed to DMP can be observed (Fig. 1 and Fig. S13, S14, ESI†). The rate constant *k* that is obtained from the integration values as a function of time fitted to a first-order decay function gives values of *k* = 3.04 (±0.34) × 10^−4^ min^−1^ ({Gd_3_(HPO_3_)Sb_3_W_28_}) (Fig. S20, ESI†), *k* = 4.46 (±0.26) × 10^−4^ min^−1^ ({Y_3_(HPO_3_)Sb_3_W_28_}) (Fig. S21, ESI†), and *k* = 7.79 (±0.46) × 10^−4^ min^−1^ ({Yb_3_(HPO_3_)Sb_3_W_28_}) (Fig. S22, ESI†). Given their isostructural nature, the observed activity trend can be related to the nature of the incorporated Lewis-acid metal centers suggesting an increasing Lewis-acid activity with decreasing ionic radius^22^{Gd_3_(HPO_3_)Sb_3_W_28_} < {Y_3_(HPO_3_)Sb_3_W_28_} < {Yb_3_(HPO_3_)Sb_3_W_28_} (Fig. S29, ESI†).

**Fig. 1:**
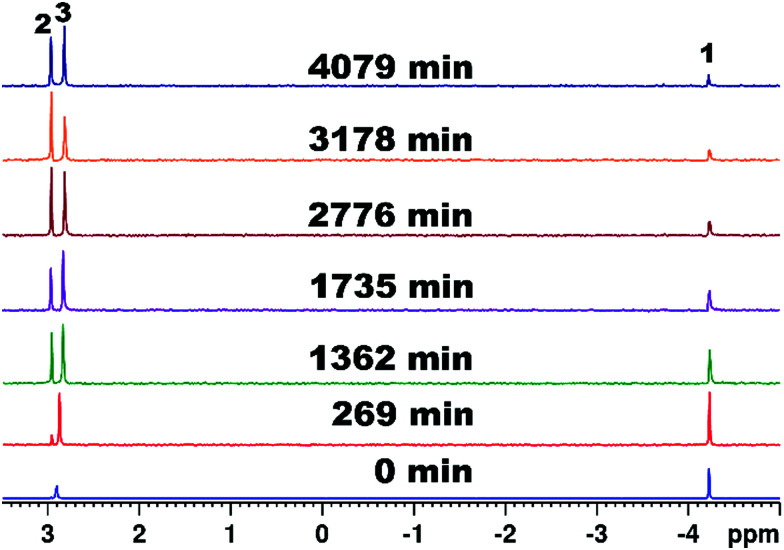
^31^P NMR spectra of the hydrolysis of DMNP (1) [4.2 mM] to nitrophenol (NP) and dimethyl phosphate (DMP) (2) with {Y_3_(HPO_3_)Sb_3_W_28_} [2.5 mM] in Tris–HCl [125 mM] at pD 7.0 25 °C. The virtually unchanged singlet peak at 2.81 ppm corresponds to the {HPO_3_} group (3) incorporated in the diamagnetic representative {Y_3_(HPO_3_)Sb_3_W_28_} suggesting solution stability of the isostructural series under turnover conditions.

Control experiments featuring the unsubstituted tungstoantimonate POM lacunary building block {SbW_9_} under elsewise identical reaction conditions (125 mM Tris–HCl, pD 7) revealed catalytic activity with a rate constant of *k* = 5.10 (±0.35) × 10^−4^ min^−1^ (Fig. S15 and S23, ESI†). To explore the unprecedented activity of the POM lacunary building block, experiments featuring varying Tris–HCl buffer concentrations were performed revealing an increase of the substrate conversion with increasing buffer concentration amounting to up to a 1.7-fold substrate conversion (93.4% in 500 mM Tris–HCl, pD 7 vs 54.4% in 125 mM Tris–HCl, pD 7) as compared to the turnover conditions chosen to probe the TAs’ phosphoesterase activity (125 mM Tris–HCl, pD 7) (Fig. 2), whereas experiments lacking any POM revealed virtually no conversion. Comparative hydrolysis studies in absence of any buffer (25 °C in D_2_O, pD = 7.0 *via* NaOD) revealed a dramatic drop in the reactivity of {SbW_9_} (*k* = 5.63 (±0.57) × 10^−5^ min^−1^) (Fig. S19 and S27, ESI†). To exclude the increasing ionic strength with increasing buffer concentrations as a reason for the observed change in catalytic performance,^23^ control experiments applying reaction mixtures with varying ionic strengths (adjusted by addition of 100–300 mM NaCl, pD 7 at 25 °C) in absence of Tris were performed thereby revealing a negligible change in substrate conversion for {SbW_9_} (8.3% in the presence of 100 mM NaCl vs 8.8% in the presence of 300 mM NaCl) (Fig. S40, ESI†). In contrast, an increase of the Lewis-acid substituted TAs’ {M_3_(HPO_3_)Sb_3_W_28_} catalytic activity by up to a factor of 2.5 can be observed under buffer-free conditions (D_2_O, pD 7 *via* NaOD at 25 °C) amounting to the same activity trend found for buffered conditions {Gd_3_(HPO_3_)Sb_3_W_28_} (*k* = 7.97 (±0.53) × 10^−4^ min^−1^) (Fig. S16 and S24, ESI†) < {Y_3_(HPO_3_)Sb_3_W_28_} (*k* = 9.23 (±0.40) × 10^−4^ min^−1^) (Fig. S17 and S25, ESI†) < {Yb_3_(HPO_3_)Sb_3_W_28_} (*k* = 12.79 (±0.38) × 10^−4^ min^−1^) (Fig. S18 and S26, ESI†). With a maximum rate constant of *k* = 12.79 (±0.38) × 10^−4^ min^−1^{Yb_3_(HPO_3_)Sb_3_W_28_} is the fastest TA-representative out of the series and exhibits a comparable catalytic performance relative to previously reported Lewis-acid containing homogeneous POM-based phosphoesterases such as [(Al_4_(H_2_O)_10_(*β*-SbW_9_O_33_)_2_]^6−^ (*k* = 4.14 × 10^−4^ min^−1^ at pD 7),^24^ [Ga_4_(H_2_O)_10_(*β*-SbW_9_O_33_)_2_]^6−^ (*k* = 2.81 × 10^−4^ min^−1^ at pD 7),^25^ and [Zr_4_(P_2_W_16_O_59_)_2_(*μ*_3_-O)_2_(OH)_2_(H_2_O)_4_]^14−^ (*k* = 5.06 × 10^−3^ min^−1^ at pD 6.4, 50 °C).^26^

**Fig. 2:**
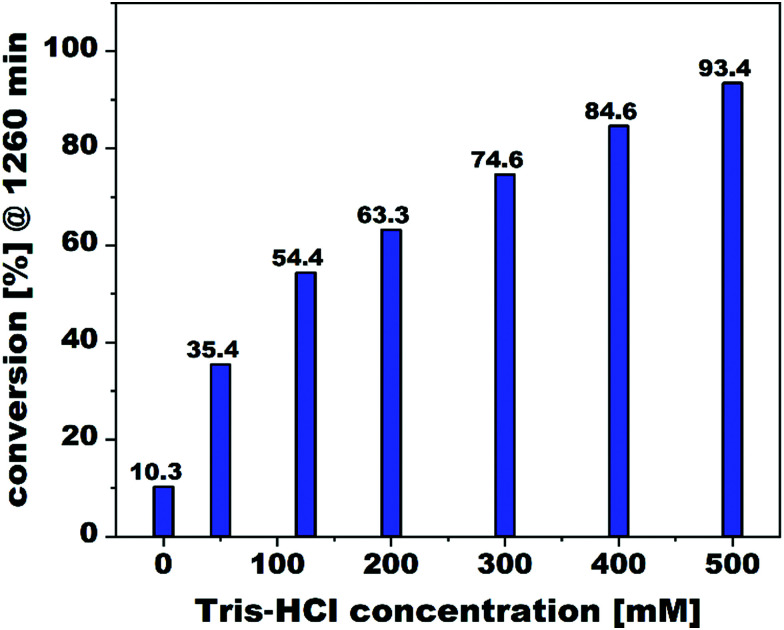
Comparison of the DMNP conversion (Scheme S1, ESI†) by the unsubstituted {SbW_9_} lacunary POM catalyst after 1260 min of incubation upon increasing the Tris–HCl buffer concentration under elsewise identical reaction conditions (pD = 7.0, 25 °C).

Considering the strong basic nature of the terminal O-centers present at the vacant sites of {SbW_9_}, an explanation for the observed rate-enhancing effect of the applied Tris-buffer system on the TA's catalytic activity would be the {SbW_9_}-mediated deprotonation of Tris, which in turn acts as a proximal base for the activation of H_2_O resulting in the generation of a OH^−^ nucleophile (Scheme S2, ESI†). The proposed non-innocence of Tris as a proximal base is in accordance with previously reported observations of the buffer non-innocence of acetate^20^ and also applies to other trilacunary lone-pair containing polyoxotungstates as shown by control experiments featuring the compositionally and structurally similar arsenotungstate (AT) Na_9_[*B-α*-AsW_9_O_33_]·27H_2_O {AsW_9_}^27^ revealing almost identical catalytic activity for the AT (Fig. S41, ESI†).

In stark contrast to the rate-enhancing effect of Tris on the catalytic activity of {SbW_9_}, the observed inhibitory effect on the activity of {M_3_(HPO_3_)Sb_3_W_28_} (Fig. S39, ESI†) can be explained by the increasing ionic strength at higher buffer concentrations considering that Lewis-acidic POM-based phosphoesterases generally follow a different mechanism suggesting the DMNP substrate's coordination to the Lewis-acid metal centers present in the 4f- and 4d-substituted TAs and subsequent hydrolysis by the OH^−^ nucleophile that is generated by activation of H_2_O coordinated to the Lewis-metal centers.^28^ The proposed mechanism of action for {M_3_(HPO_3_)Sb_3_W_28_} is in accordance with previously reported reaction mechanisms of POM-based Lewis-acids acting as phosphoesterases and additionally supported by the observed catalytic activity trend as a function of the incorporated Lewis-acid metal center (Fig. S29, ESI†) pointing towards the incorporated 4f- or 4d-metal ions to be the active metal sites.

A broad range of ^31^P NMR spectroscopic measurements (Fig. S12, S28 and S34, ESI†), post-catalytic IR-spectroscopic – (Fig. S30–S32, ESI†) and energy dispersive X-ray (EDX) analyses (Fig. S33, ESI†), as well as re-loading experiments (Fig. S34–S38 and Table S14, ESI†) elaborately discussed in the ESI† unequivocally demonstrates the stability, integrity, and recyclability of {M_3_(HPO_3_)Sb_3_W_28_} and {SbW_9_} as homogeneous phosphoesterases.

The present study demonstrates the double-template approach as a versatile synthetic tool to fine-tune the incorporated Lewis-acid metal center as a well-defined active site of the isostructural TA-series {M_3_(HPO_3_)Sb_3_W_28_} (M = Gd^III^, Y^III^, Yb^III^, *n* = 35–36). Apart from the nature of the incorporated active metal site directly correlating with the phosphoesterase activity of the resulting {M_3_(HPO_3_)Sb_3_W_28_} representative, careful attention should be given to the choice of the buffer system considering the non-innocence of Tris rendering the unsubstituted trilacunary {SbW_9_} a homogeneous phosphoesterase with activity comparable to that of the substituted counterparts. The catalytic activity of {SbW_9_} through a reaction mechanism thought to differ from the conventionally known Lewis acidic POM phosphoesterase pathway underscores the potential of lacunary POMs as phosphoesterases beyond their traditional use as molecular building blocks. The recyclability, affordable nature, and well-defined speciation chemistry of the herein reported substituted and unsubstituted POM-based phosphoesterases encourage their immobilization on matrices to yield materials combining the well-defined nature of homogeneous catalysts with the high surface area and catalytic activity of their heterogeneous counterparts as a subject of future studies.

This paper is dedicated to Prof. Dr Karl Wieghardt on the occasion of his 80th birthday. The research was funded by the Austrian Science Fund (FWF): P33089 (to A. R.) and the University of Vienna. Nuclear magnetic resonance (NMR) measurements were performed by Susanne Felsinger, Ricarda Ofenschüssl, and Sabine Schneider at the NMR core facility, Faculty of Chemistry, University of Vienna. The authors thank Mag. Johannes Theiner (Mikroanalytisches Laboratorium, Universität Wien) for elemental analyses and Ing. Claudia Mitterer (Department of Materials Chemistry, Faculty of Chemistry, University of Vienna) for experimental assistance with TGA measurements. EDX measurements were performed with great support by Assoc. Prof. Jia Min Chin, Ph. D (Department of Inorganic Chemistry – functional materials) and Assoc. Prof. Mag. Dr Michael Reithofer (Department of Inorganic Chemistry). Lastly, the authors wish to thank Ing. Dipl.-Ing. (FH) Alexander Prado-Roller (X-ray Structure Analysis Center, Faculty of Chemistry, University of Vienna) for support and valuable discussions with single crystal X-ray crystallographic measurements and data analyses.

## Conflicts of interest

There are no conflicts of interest.

## Supplementary Material

CC-058-D2CC02223K-s001

CC-058-D2CC02223K-s002
