# Supplementary material for: Trisubstituted 4f- and 4d tungstoantimonates as artificial phosphoesterases for nerve agent degradation
Source: Chem Commun (Camb). 2022 Jun 13;58(56):7761–4. doi: 10.1039/d2cc02223k (PMC9275748; doi:10.1039/d2cc02223k)
Supplement: CC-058-D2CC02223K-s001 [file CC-058-D2CC02223K-s001.pdf]

Electronic Supplementary Material (ESI) for Chemical Communications.
This journal is © The Royal Society of Chemistry 2022

ELECTRONIC SUPPLEMENTARY INFORMATION

Trisubstituted 4f – and 4d Tungstoantimonates as artificial Phosphoesterases for Nerve Agent Degradation

Elias Tanuhadi† and Annette Rompel*,†

*correspondence to annette.rompel@univie.ac.at

†Universität Wien, Fakultät für Chemie, Institut für Biophysikalische Chemie, Josef-Holaubek-Platz 2, 1090 Wien, Austria. www.bpc.univie.ac.at

Content

1. General Information 3

2. Experimental Procedure 4

2.1. Preparation of $K_3Na_{21}[(Gd(CH_3COO))_3(HPO_3)(WO_4)(SbW_9O_{33})_3]$ • 35 H_2O **{$Gd_3(HPO_3)Sb_3W_{28}$}** 4

2.2. Preparation of $K_3Na_{21}[(Y(CH_3COO))_3(HPO_3)(WO_4)(SbW_9O_{33})_3]$ • 36 H_2O **{$Y_3(HPO_3)Sb_3W_{28}$}** 4

2.3. Preparation of $K_3Na_{21}[(Yb(CH_3COO))_3(HPO_3)(WO_4)(SbW_9O_{33})_3]$ • 36 H_2O **{$Yb_3(HPO_3)Sb_3W_{28}$}** 4

3. IR-spectra 6

4. Thermogravimetric Analysis 8

5. Single-Crystal X-ray Diffraction 11

6. Powder X-ray Diffraction 16

7. UV-Vis Spectroscopy 17

8. NMR spectroscopy 18

8.1. Pre-catalytic studies 18

8.2. Post-catalytic stability studies 36

8.3. Recyclability 40

8.4. Control experiment employing a trilacunary lone-pair containing POT 46

9. References 48

1. General Information

All reagents and chemicals were of high-purity grade and were used as purchased without further purification. $Na_9[B\text{-}SbW_9O_{33}]$ • 19.5 H_2O **{SbW_9}** was prepared according to a literature procedure and characterized employing IR-spectroscopy and XRD.[1] $Na_9[B\text{-}\alpha\text{-}AsW_9O_{33}]\cdot 27H_2O$ **{AsW_9}** was prepared according to the reported literature.[2]

Elemental analysis: Elemental microanalysis of C/H/N/O/P contents was performed by Mikroanalytisches Laboratorium (University Vienna, Faculty of Chemistry). An EA 3000 (Eurovector) was used for C/H/N/S-analysis. O-determination was performed by high temperature digestion using the HT 1500 (Hekatech, Germany) pyrolysis system in combination with the EA 3000 system.

Attenuated total reflection Fourier−transform Infrared Spectroscopy: All spectra were recorded on a Bruker Vertex70 IR Spectrometer equipped with a single−reflection diamond−ATR unit. Frequencies are given in cm^{-1}, intensities denoted as w = weak, m = medium, s = strong.

Thermogravimetric analysis (TGA): was conducted at the Department of Materials Chemistry, Faculty of Chemistry, University of Vienna, using a thermal analyzer (TA instruments model Q50, USA). Each sample having an initial mass of ~15 mg was subjected to a temperature range of 298 - 1123 K at a heating rate of 5 K min^{-1}.

Single crystal X-ray diffraction (SXRD): The X-ray data were measured on a Bruker D8 Venture equipped with a multilayer monochromator, *MoK/α* (λ = 0.71073Å) INCOATEC micro focus sealed tube (**{$Y_3(HPO_3)Sb_3W_{28}$}**) and a STOE Stadivari dual source (Mo radiation), equipped with a Dectris EIGER2 R500 detector (**{$Gd_3(HPO_3)Sb_3W_{28}$}**, and **{$Yb_3(HPO_3)Sb_3W_{28}$}**, **Na{$Y_3(HPO_3)Sb_3W_{28}$}**), respectively. The structures were solved by direct methods and refined by full-matrix least-squares. Non-hydrogen atoms were refined with anisotropic displacement parameters. The following software was used for the structure-solving procedure: frame integration, Bruker SAINT software package using a narrow-frame algorithm (absorption correction)[3], SADABS[4], SHELXS-2013[5] and X-area (structure solution), SHELXL-2013[6] (refinement), OLEX2[7] (structure solution, refinement, molecular diagrams, and graphical user-interface), and SHELXLE[8] (molecular diagrams and graphical user interface). Experimental data and CCDC-codes are provided in **Tables S5-S13**.

Powder X-ray diffraction was performed at the Department of Inorganic Chemistry, Faculty of Chemistry, University of Vienna employing a Bruker D8 ADVANCE diffractometer, Cu*Kα* radiation, λ = 1.54056 Å, LYNXEYE silicon strip detector and SolX energy dispersive detector, variable slit aperture with 12 mm, 8° ≤ 2θ ≤ 50°.

Energy dispersive X-ray (EDX) analysis data were obtained at Department of Inorganic Chemistry - functional materials, Institute of Chemistry, University of Vienna using an Oxford Instruments INCA Energy SEM-EDS system.

UV/Vis spectroscopy: UV/Vis spectra were collected on a Shimadzu UV 1800 spectrophotometer.

2. Experimental Procedure

{$M_3(HPO_3)Sb_3W_{28}$} were prepared employing a double-template synthetic strategy, which, in contrast to conventional template approaches exploits more than one template agent to yield more complex POM architectures exhibiting modulable properties depending on the type and number of incorporated template.[9] In the present study, a {HPO_3} motif was incorporated into the POM framework in addition to the stabilizing central tetrahedral {WO_4} moiety thereby allowing ^{31}P NMR spectroscopy experiments for speciation studies under catalytic turnover conditions.

2.1. Preparation of $K_3Na_{21}[(Gd(CH_3COO))_3(HPO_3)(WO_4)(SbW_9O_{33})_3]$ • 35 H_2O {$Gd_3(HPO_3)Sb_3W_{28}$}

To a stirred heated suspension (~80°C) of Na_9[*B*-*α*-SbW_9O_{33}]·19.5 H_2O (3.500 g, 1.222 mmol), Na_2WO_4·2H_2O (0.200 g, 0.607 mmol) and KCl (0.100 g, 1.341 mmol) in 15 mL of a mixture of 10 mL NaOAc/AcOH buffer (2 M, pH = 5.5) and 5 mL H_2O (T = 85 – 90 °C) H_3PO_3 (0.100 g, 1.220 mmol) and $Gd(NO_3)_3$ • 6 H_2O (0.202 g, 0.448 mmol) were successively added. After cooling to room temperature, the solution's pH was re-adjusted to 5.5 via dropwise addition of HCl [6 M] followed by room temperature stirring for 30 min and heating to 90°C for 1 h. Filtration and room temperature evaporation (T = 20°C) of the yellow reaction mixture afforded yellow block shaped crystals of **{$Gd_3(HPO_3)Sb_3W_{28}$}** after two days. Yield: 936 mg, 23% based on Gd. Anal. Calcd. (%) for $C_6H_{80}PW_{28}Sb_3O_{147}Gd_3Na_{21}K_3$ ($K_3Na_{21}[(Gd(CH_3COO))_3(HPO_3)(WO_4)(SbW_9O_{33})_3]$ • 35 H_2O): C, 0.79; H, 1.01; O, 25.79; P, 0.34; Found: C, 0.65; H, 0.88; O, 26.23; P, 0.40.

2.2. Preparation of $K_3Na_{21}[(Y(CH_3COO))_3(HPO_3)(WO_4)(SbW_9O_{33})_3]$ • 36 H_2O {$Y_3(HPO_3)Sb_3W_{28}$}

To a stirred heated suspension (~80°C) of Na_9[*B*-*α*-SbW_9O_{33}]·19.5 H_2O (3.500 g, 1.222 mmol), Na_2WO_4·2 H_2O (0.200 g, 0.607 mmol) and KCl (0.100g, 1.341 mmol) in 15 mL of a mixture of 10 mL NaOAc/AcOH buffer (2 M, pH = 5.5) and 5 mL H_2O (T = 85 – 90 °C) H_3PO_3 (0.100 g, 1.220 mmol) and YCl_3 • H_2O (0.138 g, 0.448 mmol) were successively added. After cooling to room temperature, the solution's pH was re-adjusted to 5.5 via dropwise addition of HCl [6 M] followed by room temperature stirring for 30 min and heating to 90°C for 1 h. Filtration and room temperature evaporation (T = 20°C) of the yellow reaction mixture afforded yellow block shaped crystals of **{$Y_3(HPO_3)Sb_3W_{28}$}** after two days. Yield: 940 mg, 23.5% based on Y. Anal. Calcd. (%) for $C_6H_{82}PW_{28}Sb_3O_{148}Y_3Na_{21}K_3$ ($K_3Na_{21}[(Y(CH_3COO))_3(HPO_3)(WO_4)(SbW_9O_{33})_3]$ • 36 H_2O): C, 0.81; H, 0.93; O, 26.51; P, 0.35; K, 1.31; Na, 5.40; Found: C, 0.81; H, 0.94; O, 26.50; P, 0.35; K, 1.31; Na, 5.40.

2.3. Preparation of $K_3Na_{21}[(Yb(CH_3COO))_3(HPO_3)(WO_4)(SbW_9O_{33})_3]$ • 36 H_2O {$Yb_3(HPO_3)Sb_3W_{28}$}

To a stirred heated suspension (~80°C) of Na_9[*B*-*α*-SbW_9O_{33}]·19.5H_2O (3.500 g, 1.222 mmol), Na_2WO_4·2H_2O (0.200 g, 0.607 mmol) and KCl (0.100 g, 1.341 mmol) in 15 mL of a mixture of 10 mL NaOAc/AcOH buffer (2 M, pH = 5.5) and 5 mL H_2O (T = 85 – 90 °C) H_3PO_3 (0.100 g, 1.220 mmol) and $YbCl_3$ • 6 H_2O (0.173 g, 0.448 mmol) were successively added. After cooling

to room temperature, the solution's pH was re-adjusted to 5.5 via dropwise addition of HCl [6 M] followed by room temperature stirring for 30 min and heating to 90°C for 1 h. Filtration and room temperature evaporation (T = 20°C) of the yellow reaction mixture afforded yellow block shaped crystals of **{$Yb_3(HPO_3)Sb_3W_{28}$}** after two days. Yield: 946 mg, 24.5% based on Yb. Anal. Calcd. (%) for $C_6H_{82}PW_{28}Sb_3O_{148}Yb_3Na_{21}K_3$ ($K_3Na_{21}[(Yb(CH_3COO))_3(HPO_3)(WO_4)(SbW_9O_{33})_3]$ • 36 H_2O): C, 0.78; H, 0.90; O, 25.78; Found: C, 0.68; H, 0.95; O, 26.14.

3. IR-spectra

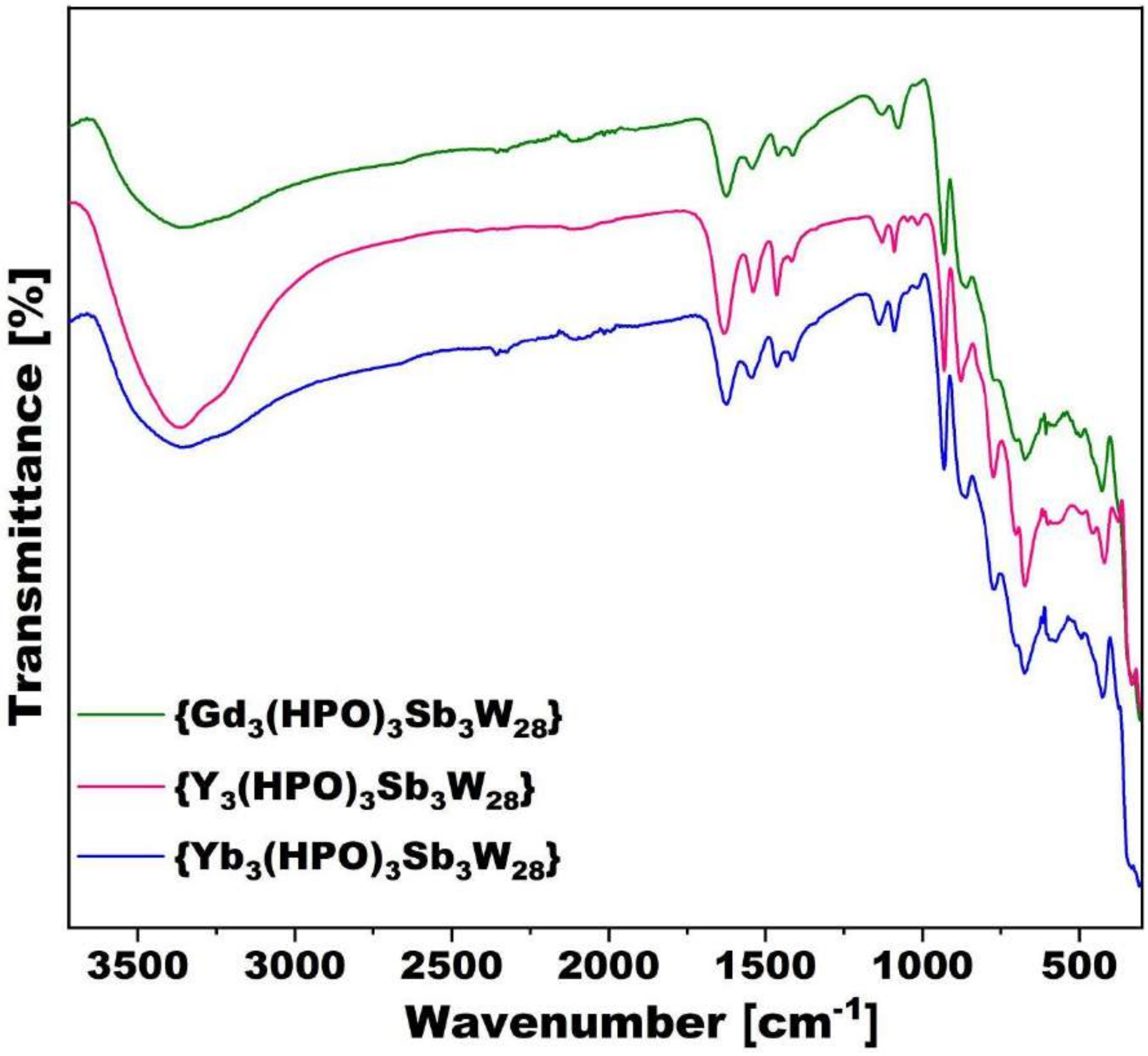

Figure S1. IR-spectra of $K_3Na_{21}[M(CH_3COO)_3(HPO_3)(WO_4)(SbW_9O_{33})_3]$ • n H_2O (n = 35 – 36) **{$M_3(HPO_3)Sb_3W_{28}$}**, M = Gd^{III} (green), Y^{III} (pink), and Yb^{III} (blue).

Table S1. Attribution and positions of the bands observed in the IR-spectra of **{$M_3(HPO_3)Sb_3W_{28}$}**. M = Gd^{III}, Y^{III}, Yb^{III}, w = weak, m = medium, s = strong. In the case of the acetate ligands, the corresponding group (methyl CH_3, or carboxylate COO^-) of which the bands arise from is highlighted in bold.

POT	position/range [cm^{-1}]	attribution	intensity
**{$Gd_3(HPO_3)Sb_3W_{28}$}**	3344.2	$^{\nu}$OH	s
	1623.9	$^{\delta}H_2O$	s
	1539	$^{\nu}CH_3$**COO^-**	s
	1460	$^{\nu}CH_3$**COO^-**	s
	1411.7	$^{\nu}CH_3$**COO^-**	m
	1344.2	$^{\delta}$**CH_3**COO^-	w
	931.5	$^{\nu}$W=O	s
	871.5	Gd-O(W)	m
	860.2 - 335	$^{\nu}$W=O, $^{\delta}$W-O-W	s
	835 - 495	$^{\nu}$Sb-O	s
**{$Y_3(HPO_3)Sb_3W_{28}$}**	3360.5	$^{\nu}$OH	s
	1633.5	$^{\delta}H_2O$	s
	1539	$^{\nu}CH_3$**COO^-**	s
	1463.8	$^{\nu}CH_3$**COO^-**	s
	1413.7	$^{\nu}CH_3$**COO^-**	m
	1342.3	$^{\delta}$**CH_3**COO^-	w
	1205.4	$^{\delta}$**CH_3**COO^-	w
	931	$^{\nu}$W=O	s
	877.5	Y-O(W)	m
	865.5 - 335	$^{\nu}$W=O, $^{\delta}$W-O-W	s
	835 - 495	$^{\nu}$Sb-O	s
**{$Yb_3(HPO_3)Sb_3W_{28}$}**	3355.7	$^{\nu}$OH	s
	1623.9	$^{\delta}H_2O$	s
	1541	$^{\nu}CH_3$**COO^-**	s
	1463.8	$^{\nu}CH_3$**COO^-**	s
	1434.9	$^{\nu}CH_3$**COO^-**	w
	1413.7	$^{\nu}CH_3$**COO^-**	m
	1342.3	$^{\delta}$**CH_3**COO^-	w
	931	$^{\nu}$W=O	s
	875.6	Yb-O(W)	m
	865.5 - 335	$^{\nu}$W=O, $^{\delta}$W-O-W	s
	835 - 495	$^{\nu}$Sb-O	s

4. Thermogravimetric Analysis

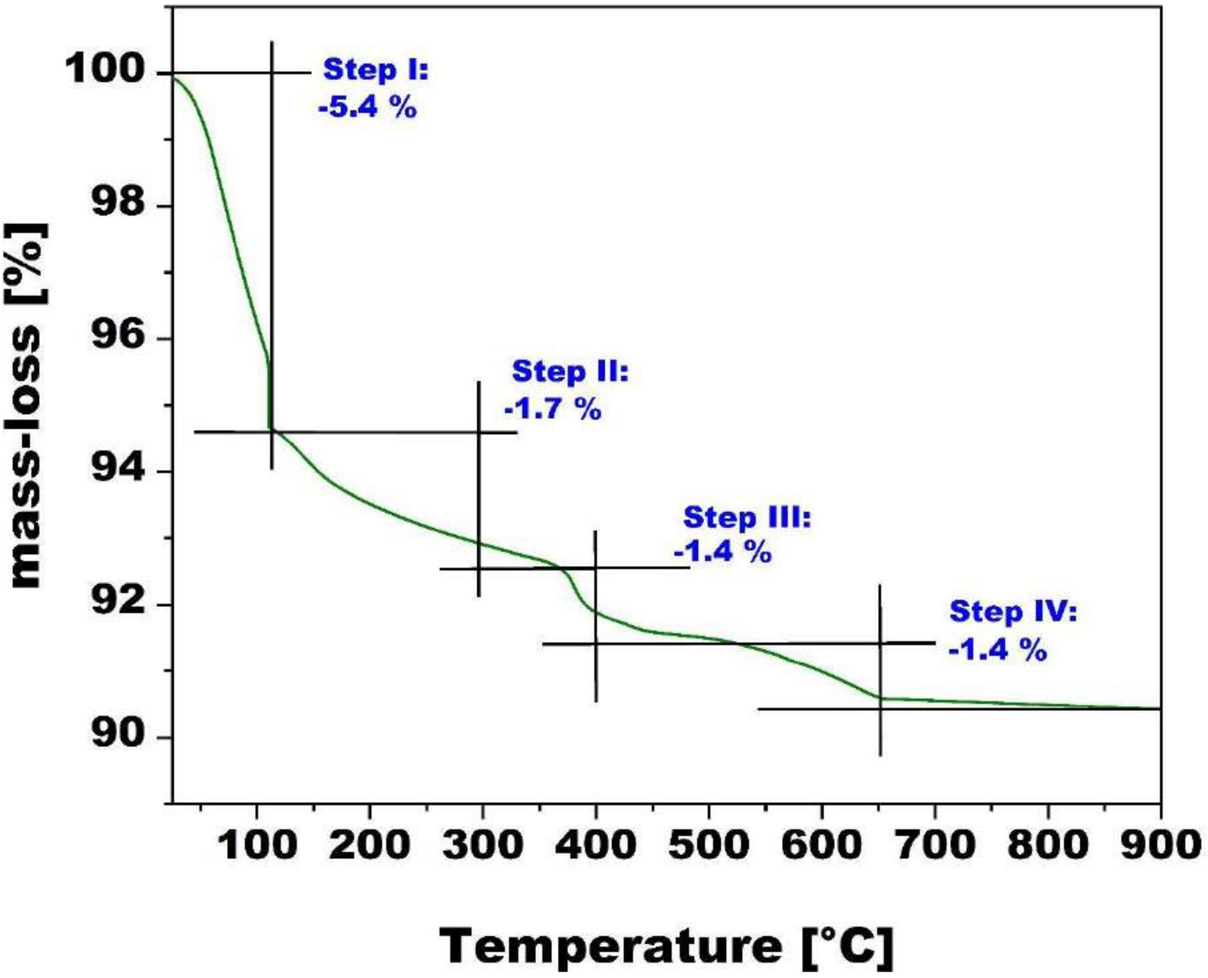

Figure S2. Thermogravimetric curve of **{$Gd_3(HPO_3)Sb_3W_{28}$}** • 35 H_2O.

Table S2. TGA results for **{$Gd_3(HPO_3)Sb_3W_{28}$}** • 35 H_2O according to the TGA curve depicted in **Figure S2**.

Step	T, °C	mass-loss, %	number of molecules corresponding to mass-loss
I	25 - 110	5.4	26 H_2O
II	111 - 370	1.7	8 H_2O
III	371 - 520	1.4	H_2O + ~1.5 CH_3COO^-
IV	521 - 900	1.4	~1.5 CH_3COO^- + POM anion decomposition

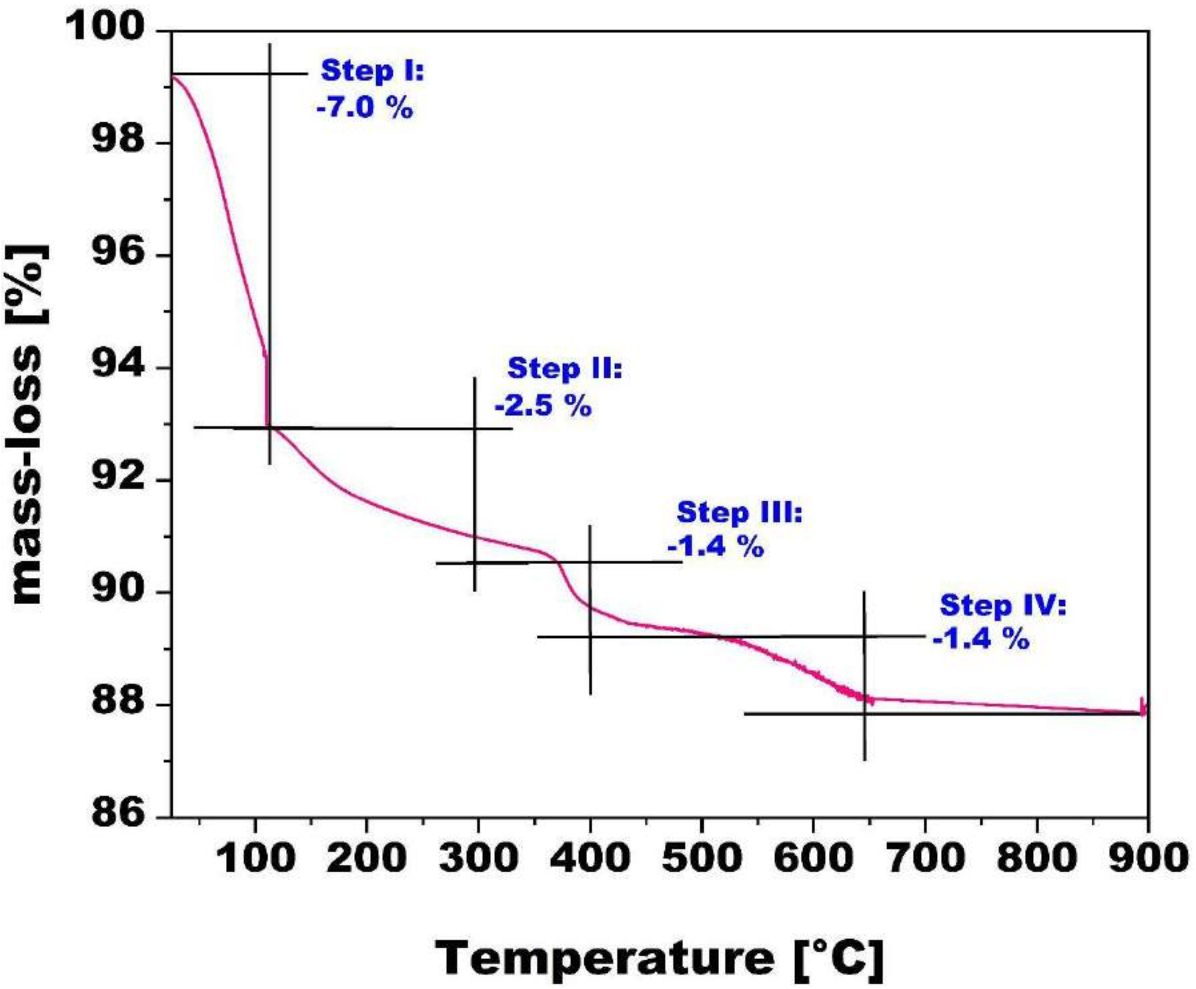

Figure S3. Thermogravimetric curve of **{$Y_3(HPO_3)Sb_3W_{28}$}** • 36 H_2O.

Table S3. TGA results for **{$Y_3(HPO_3)Sb_3W_{28}$}** • 36 H_2O according to the TGA curve depicted in **Figure S3**.

Step	T, °C	mass-loss, %	number of molecules corresponding to mass-loss
I	25 - 110	7.0	33 H_2O
II	111 - 370	2.5	3 H_2O + 2 CH_3COO^-
III	371 - 520	1.4	CH_3COO^- + anion decomposition
IV	521 - 900	1.4	POM anion decomposition

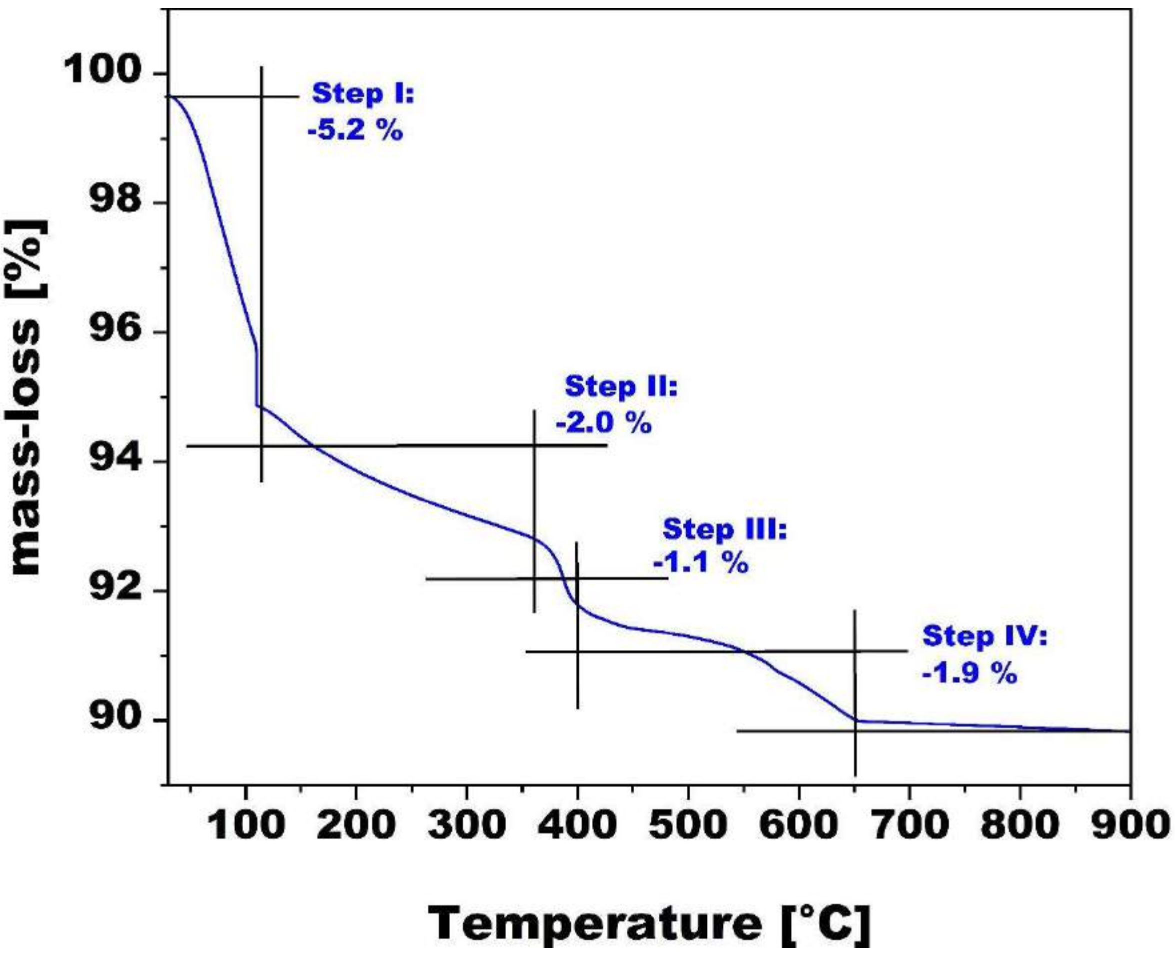

Figure S4. Thermogravimetric curve of **{$Yb_3(HPO_3)Sb_3W_{28}$}** • 36 H_2O.

Table S4. TGA results for **{$Yb_3(HPO_3)Sb_3W_{28}$}** • 36 H_2O according to the TGA curve depicted in **Figure S4**.

Step	T, °C	mass-loss, %	number of molecules corresponding to mass-loss
**I**	25 - 110	5.2	26 H_2O
**II**	111 - 370	2.0	10 H_2O
**III**	371 - 520	1.1	2 CH_3COO^-
**IV**	521 - 900	1.9	CH_3COO^- + POM-anion decomposition

5. Single-Crystal X-ray Diffraction

SXRD studies revealed that all three isostructural polyanions crystallize in the trigonal space group $R_{3/m}$ with idealized C_{3v} symmetry. The architecture of {$M_3(HPO_3)Sb_3W_{28}$} represents a trimeric polyanion comprising three trilacunary lone-pair containing $[SbW_9O_{33}]^{9-}$ units enclosing a {$(M(CH_3COO))_3(HPO_3)(WO_4)$} M = Gd^{III}, Y^{III}, Yb^{III} metal-oxo core featuring three 4d- or 4f metal ion centers with distorted square-antiprismatic coordination geometry and an average M – O bond length of 2.403(16) Å. Each 4d- or 4f- metal ion center is coordinated by an acetate-ligand and the central {HPO_3} group. The three acetate ligands can be easily exchanged and are a prerequisite for hydrolytic activity (**Scheme. 1**). A tetrahedral {WO_4} motif resides in the opposite direction to the {HPO_3} group linking to the 4d- or 4f metal centers and three charge balancing potassium counter cations, respectively.

Addition of hydrogen atoms to generate an H-bonding network with the H_2O molecules would have resulted in high shift values giving rise to an A-level alert due to the instability of the network. Considering the strong disorder of H_2O molecules preventing a satisfactory refinement, the corresponding electron densities were described employing the Olex2 solvent mask routine in the case of **{$Y_3(HPO_3)Sb_3W_{28}$}** to stabilize the refinement giving rise to a void of 735 A^3 (198 electrons), which can be attributed to 19 H_2O molecules. The quantity of H_2O molecules was determined with TGA and entered into the CIF file corresponding to the proposed sum formula, which is based on elemental analyses.

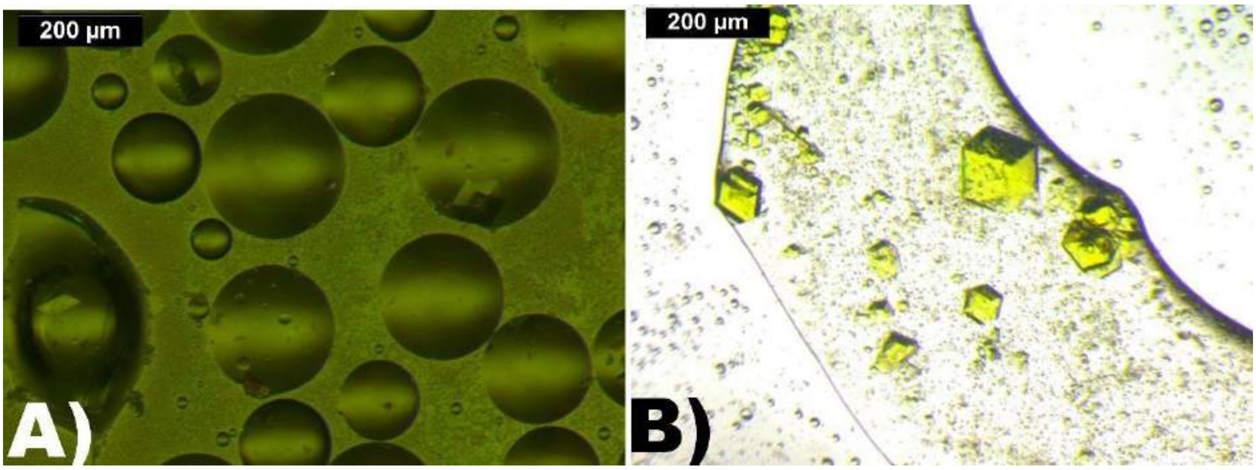

Figure S5. Pictures showing **A)** the oil that is observed upon cooling of the reaction mixture and **B)** the yellow blocks of **{$Y_3(HPO_3)Sb_3W_{28}$}** that crystallized from the oil within four days at 20°C.

Table S5. Experimental parameter and CCDC-Code. **Na{$Y_3(HPO_3)Sb_3W_{28}$}** represents **{$Y_3(HPO_3)Sb_3W_{28}$}** that was re-crystallized after pre-catalytic stability experiments have been undertaken.

Sample	Machine	Source	Temp.	CCDC
			[K]	

**{$Gd_3(HPO_3)Sb_3W_{28}$}**	STOE Stadivari	Mo	100	2167229
**{$Y_3(HPO_3)Sb_3W_{28}$}**	Bruker D8	Mo	100	2167231
**{$Yb_3(HPO_3)Sb_3W_{28}$}**	STOE Stadivari	Mo	100	2167232
**Na{$Y_3(HPO_3)Sb_3W_{28}$}**	STOE Stadivari	Mo	100	2167230

Table S6. Sample and crystal data of **{$Gd_3(HPO_3)Sb_3W_{28}$}**.

**Chemical formula**	$C_6H_{80}PW_{28}Sb_3O_{147}Gd_3Na_{21}K_3$	**Crystal system**	trigonal	
**Formula weight [g/mol]**	9120.21	**Space group**	R_{3m}	
**Temperature [K]**	100	**Z**	3	
**Measurement method**	ω scans	**Volume [Å^3]**	11749.7(11)	
**Radiation (Wavelength [Å])**	Mo Kα (λ = 0.71073)	**Unit cell dimensions [Å] and [°]**	30.6714(12)	90
**Crystal size / [mm^3]**	0.15 × 0.133 × 0.1		30.6714(12)	90
**Crystal habit**	clear yellow block		14.4221(8)	120
**Density (calculated) / [g/cm^3]**	3.867	**Absorption coefficient / [mm^{-1}]**	22.436	
**Abs. correction T_{min}**	0.0335	**Abs. correction T_{max}**	0.0776	
**Abs. correction type**	multi-scan	**F(000) [e$^-$]**	12036.0	

Table S7. Data collection and structure refinement of **{$Gd_3(HPO_3)Sb_3W_{28}$}**.

**Index ranges**	-36 ≤ h ≤ 36, -33 ≤ k ≤ 34, -17 ≤ l ≤ 17	**Theta range for data collection [°]**	4.17 to 50.684	
**Reflections number**	29428	**Data / restraints / parameters**	5006/236/410	
**Refinement method**	Least squares	**Final R indices**	all data	R_1 = 0.0569, wR_2 = 0.0909
**Function minimized**	$\Sigma\ w(F_o^2 - F_c^2)^2$		I>2σ(I)	R_1 = 0.0426, wR_2 = 0.0876
**Goodness-of-fit on F^2**	0.975	**Weighting scheme**	$w=1/[\sigma^2(F_o^2)+(0.0623P)^2]$	
**Largest diff. peak and hole [e Å^{-3}]**	1.49/-2.06		where $P=(F_o^2+2F_c^2)/3$	

Table S8. Sample and crystal data of **{$Y_3(HPO_3)Sb_3W_{28}$}**.

**Chemical formula**	$C_6H_{82}PW_{28}Sb_3O_{148}Y_3Na_{21}K_3$	**Crystal system**	trigonal	
**Formula weight [g/mol]**	8933.19	**Space group**	R_{3m}	
**Temperature [K]**	100.0	**Z**	3	
**Measurement method**	ϕ and ωscans	**Volume [Å^3]**	11881.8(6)	
**Radiation (Wavelength [Å])**	Mo*Kα* (λ = 0.71073)	**Unit cell dimensions [Å] and [°]**	30.6654(7)	90
**Crystal size / [mm^3]**	0.2 × 0.2 × 0.2		30.6654(7)	90
**Crystal habit**	clear yellow block		14.5899(4)	120
**Density (calculated) / [g/cm^3]**	3.746	**Absorption coefficient / [mm^{-1}]**	22.080	
**Abs. correction T_{min}**	0.3611	**Abs. correction T_{max}**	0.6056	
**Abs. correction type**	multi-scan	**F(000) [e$^-$]**	11841.0	

Table S9. Data collection and structure refinement of **{$Y_3(HPO_3)Sb_3W_{28}$}**.

**Index ranges**	-37 ≤ h ≤ 37, -37 ≤ k ≤ 37, -18 ≤ l ≤ 17	**Theta range for data collection [°]**	4.148 to 52.022	
**Reflections number**	32142	**Data / restraints / parameters**	5482/97/361	
**Refinement method**	Least squares	**Final R indices**	all data	R_1 = 0.0415, wR_2 = 0.1058
**Function minimized**	$\Sigma\, w(F_o^2 - F_c^2)^2$		I>2σ(I)	R_1 = 0.0401, wR_2 = 0.1048
**Goodness-of-fit on F^2**	1.055	**Weighting scheme**	$w=1/[\sigma^2(F^2)+(0.0665P)^2+385.7952P_o]$	
**Largest diff. peak and hole [e Å^{-3}]**	1.96/-1.15		where $P=(F_o^2+2F_c^2)/3$	

Table S10. Sample and crystal data of **{$Yb_3(HPO_3)Sb_3W_{28}$}**.

**Chemical formula**	$C_6H_{82}PW_{28}Sb_3O_{148}Yb_3Na_{21}K_3$	**Crystal system**	trigonal	
**Formula weight [g/mol]**	9185.60	**Space group**	R_{3m}	
**Temperature [K]**	100	**Z**	3	
**Measurement method**	ω scans	**Volume [Å^3]**	11704.3(6)	
**Radiation (Wavelength [Å])**	Mo K_α (λ = 0.71073)	**Unit cell dimensions [Å] and [°]**	30.5334(6)	90

**Crystal size / [mm³]**	0.2 × 0.2 × 0.2		30.5334(6)	90
**Crystal habit**	clear yellow block		14.4965(4)	120
**Density (calculated) / [g/cm³]**	3.902	**Absorption coefficient / [mm⁻¹]**	23.090	
**Abs. correction T_{min}**	0.0025	**Abs. correction T_{max}**	0.0129	
**Abs. correction type**	multi-scan	**F(000) [e⁻]**	12090.0	

Table S11. Data collection and structure refinement of **{$Yb_3(HPO_3)Sb_3W_{28}$}**.

**Index ranges**	-36 ≤ h ≤ 36, -36 ≤ k ≤ 36, -17 ≤ l ≤ 17	**Theta range for data collection [°]**	4.17 to 49.422	
**Reflections number**	160304	**Data / restraints / parameters**	4704/1039/ 454	
**Refinement method**	Least squares	**Final R indices**	all data	R_1 = 0.0584, wR_2 = 0.1134
**Function minimized**	$\Sigma w(F_o^2 - F_c^2)^2$		I>2σ(I)	R_1 = 0.0456, wR_2 = 0.1092
**Goodness-of-fit on F^2**	0.967	**Weighting scheme**	$w=1/[\sigma^2(F_o^2)+(0.0895P)^2]$	
**Largest diff. peak and hole [e Å⁻³]**	1.92/-1.77		where $P=(F_o^2+2F_c^2)/3$	

Table S12. Data collection and structure refinement of **Na{$Y_3(HPO_3)Sb_3W_{28}$}**.

**Chemical formula**	$C_6H_{82}PW_{28}Sb_3O_{148}Y_3Na_{21}K_3$	**Crystal system**	trigonal	
**Formula weight [g/mol]**	8933.19	**Space group**	R_{3m}	
**Temperature [K]**	100	**Z**	3	
**Measurement method**	ω scans	**Volume [Å³]**	11822.9(6)	
**Radiation (Wavelength [Å])**	Mo K_α (λ = 0.71073)	**Unit cell dimensions [Å] and [°]**	30.5863(5)	90
**Crystal size / [mm³]**	0.18 × 0.18 × 0.18		30.5863(5)	90
**Crystal habit**	clear yellow block		14.5855(3)	120
**Density (calculated) / [g/cm³]**	3.764	**Absorption coefficient / [mm⁻¹]**	22.190	
**Abs. correction Tmin**	0.5616	**Abs. correction Tmax**	0.7536	
**Abs. correction type**	multi-scan	**F(000) [e⁻]**	11841.0	

Table S13. Sample and crystal data of **Na{$Y_3(HPO_3)Sb_3W_{28}$}**.

**Index ranges**	-30 ≤ h ≤ 35, -35 ≤ k ≤ 21, -17 ≤ l ≤ 17	**Theta range for data collection [°]**	4.154 to 49.426	
**Reflections number**	24569	**Data / restraints / parameters**	4676/294/384	
**Refinement method**	Least squares	**Final R indices**	all data	R_1 = 0.0539, wR_2 = 0.1171
**Function minimized**	$\Sigma\ w(F_o^2 - F_c^2)^2$		I>2σ(I)	R_1 = 0.0452, wR_2 = 0.1115
**Goodness-of-fit on F^2**	1.017	**Weighting scheme**	$w=1/[\sigma^2(F_o^2)+(0.0894P)^2]$	
**Largest diff. peak and hole [e Å^{-3}]**	2.59/-3.31		where $P=(F_o^2+2F_c^2)/3$	

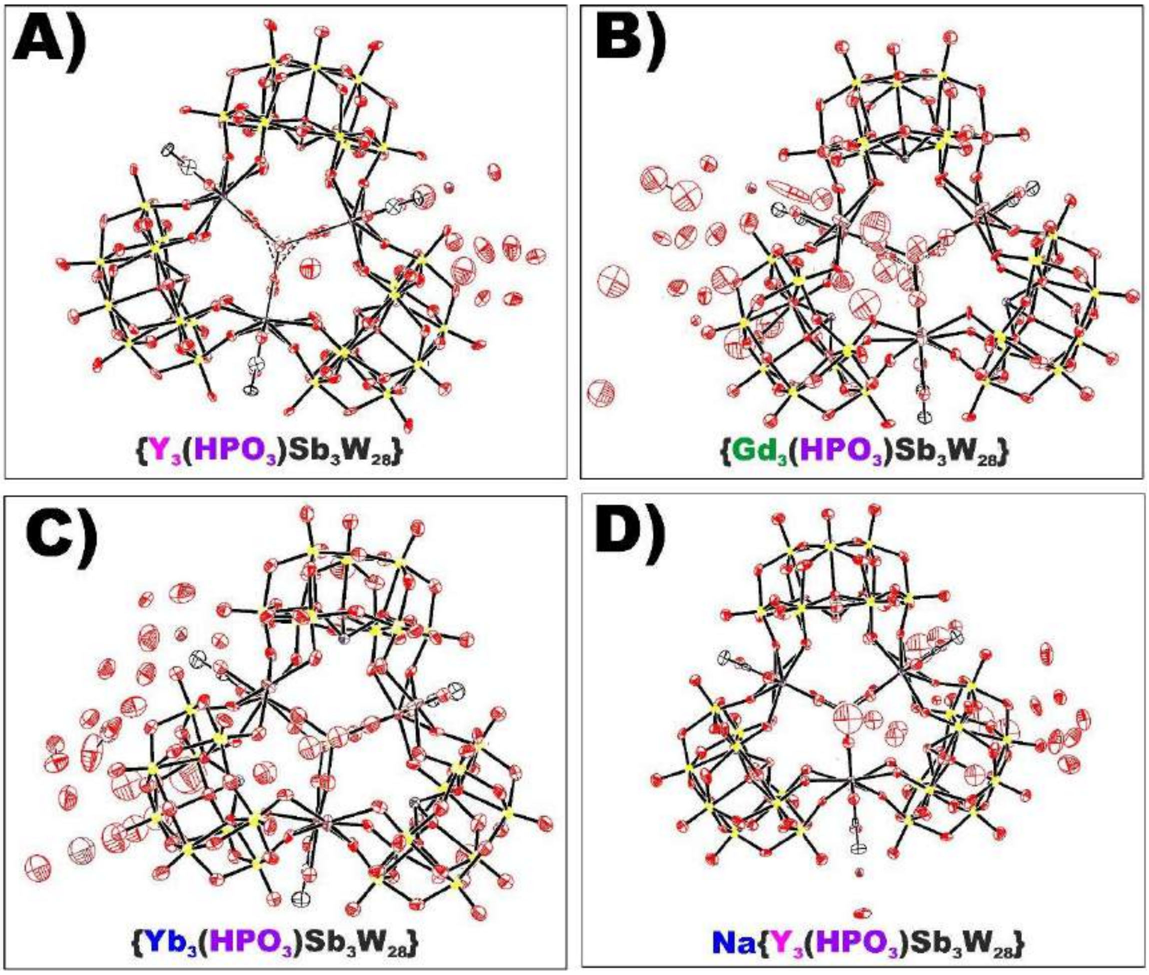

Figure S6. ORTEP showing atomic displacement parameters (ADPs) of **A) {$Y_3(HPO_3)Sb_3W_{28}$}**, **B) {$Gd_3(HPO_3)Sb_3W_{28}$}**, **C) {$Yb_3(HPO_3)Sb_3W_{28}$}**, and **D) Na{$Y_3(HPO_3)Sb_3W_{28}$}**.

6. Powder X-ray Diffraction

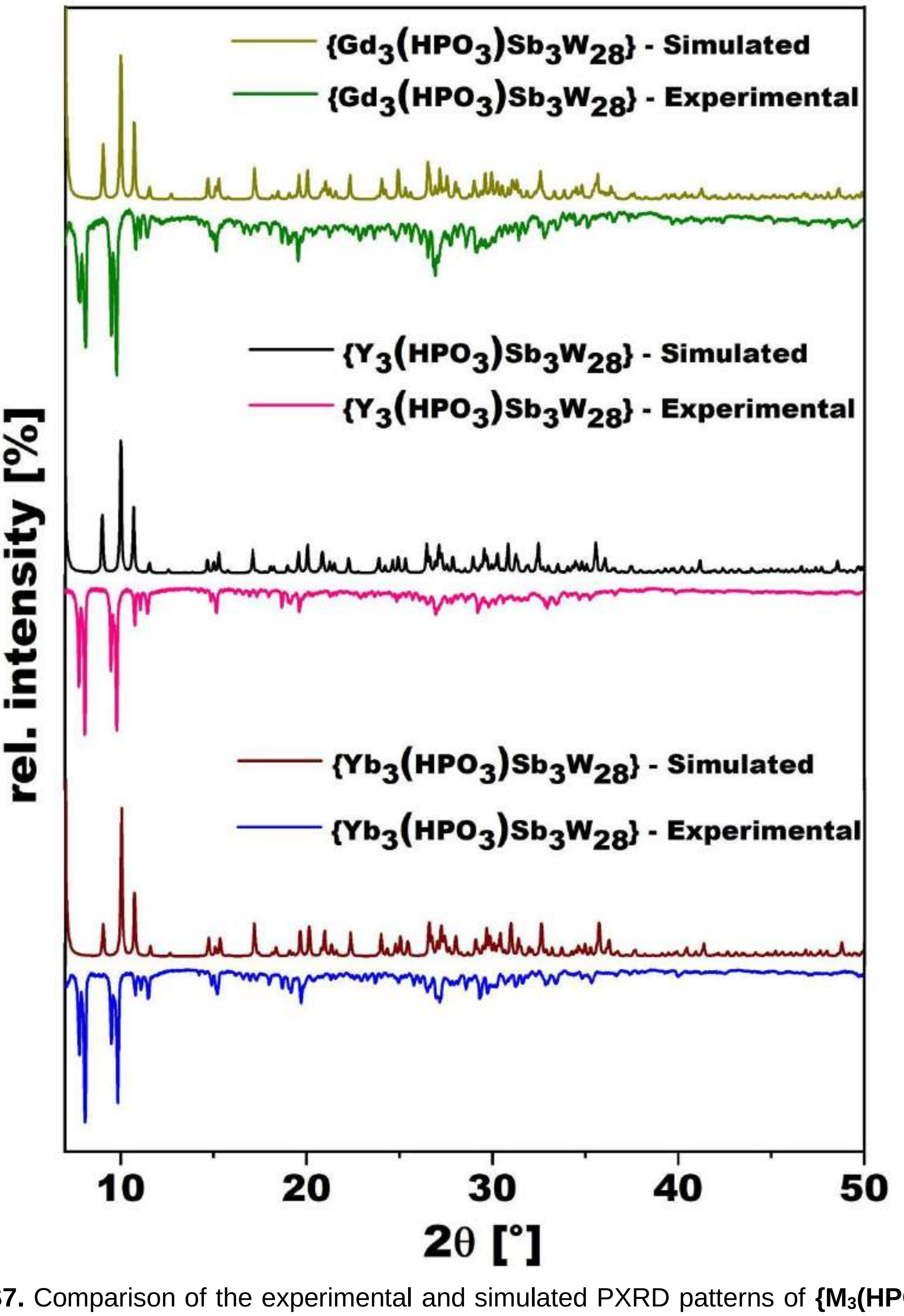

Figure S7. Comparison of the experimental and simulated PXRD patterns of **{$M_3(HPO_3)Sb_3W_{28}$}**. Note that differences between the simulated and the experimental PXRD patterns may be due to factors such as loss of solvent molecules as indicated by TGA measurements further leading to the collapse of the lattice giving rise to different crystalline phases at different temperatures. Additionally, the experimental PXRD pattern was obtained at room temperature (298 K), whereas the CIF file used for the simulated pattern was obtained from a single crystal measured at 112 K.

7. UV-Vis Spectroscopy

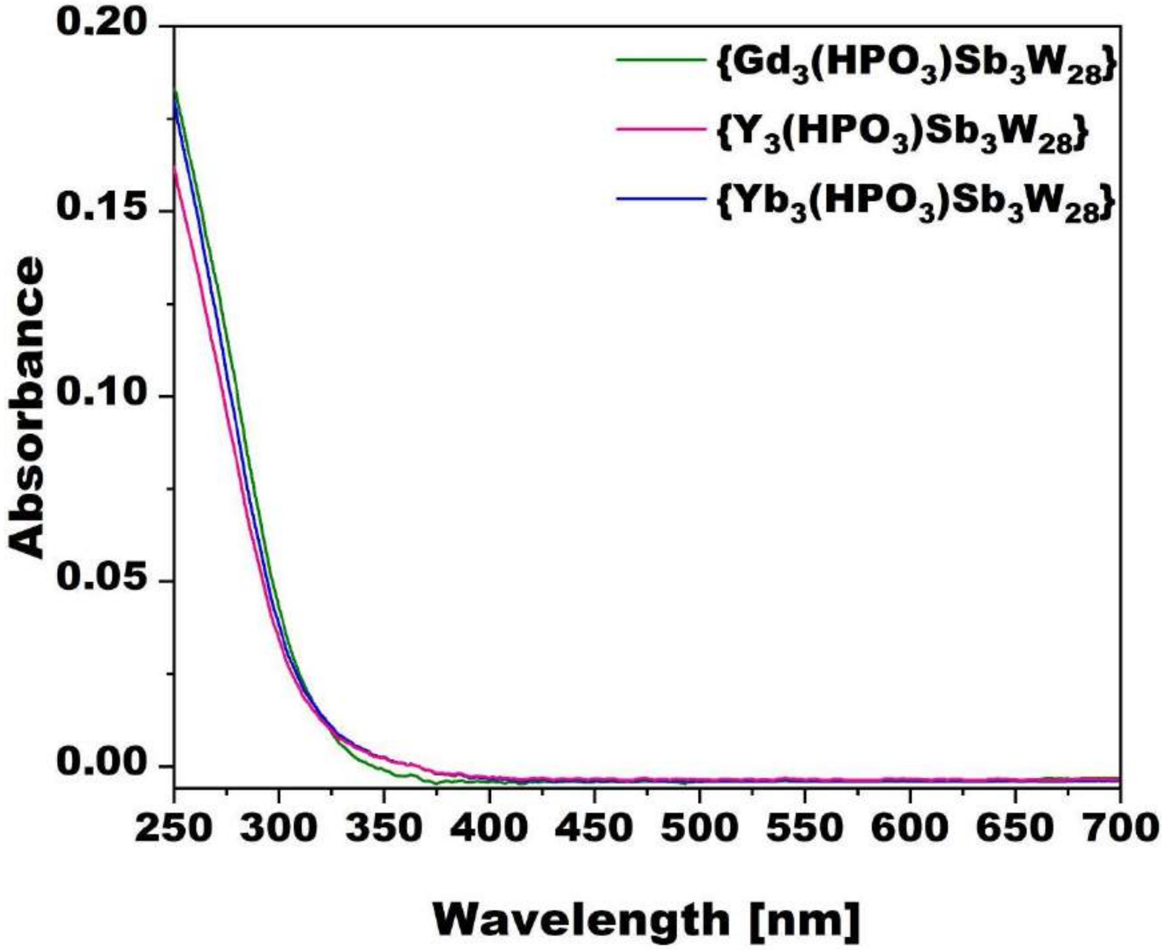

Figure S8. UV/Vis-spectra of **{$Gd_3(HPO_3)Sb_3W_{28}$}** [7.5 μM], **{$Y_3(HPO_3)Sb_3W_{28}$}** [7 μM], and **{$Yb_3(HPO_3)Sb_3W_{28}$}** [9 μM] in D_2O, pH = 7.0 displaying an absorption maximum at ~250 nm.

8. NMR spectroscopy

8.1. Pre-catalytic studies

The ^{13}C NMR spectrum of a freshly prepared aqueous solution of **{$Y_3(HPO_3)Sb_3W_{28}$}** displays two peaks corresponding to the methyl - (δ = 23.20 ppm) and carboxyl group (δ = 181.46 ppm), respectively (**Fig. S9**). Subsequent addition of NaOAc to the **{$Y_3(HPO_3)Sb_3W_{28}$}** solution resulted in an increase of both ^{13}C NMR peaks with no additionally occurring peaks, thereby suggesting the exclusive presence of free acetate ligands in aqueous solution, hence demonstrating the facilitated accessibility of the Lewis-acid metal centers in **{$M_3(HPO_3)Sb_3W_{28}$}** (**Fig. S10 A**, **B**).

The ^{31}P NMR spectrum of **{$Y_3(HPO_3)Sb_3W_{28}$}** in D_2O (pD = 7.0) displays a singlet peak located at 2.73 ppm which can be attributed to the central {HPO_3} motif present in all isostructural **TA**s of the compound series (**Fig. S11 A, B**). In contrast, the ^{31}P NMR spectrum of a freshly prepared aqueous solution of phosphorous acid displays various peaks (**Fig. S11 A**) thereby excluding the presence of free phosphorous acid in the case of **{$M_3(HPO_3)Sb_3W_{28}$}**. Ageing experiments that were performed by incubating a freshly prepared aqueous solution of **{$Y_3(HPO_3)Sb_3W_{28}$}** under pre-catalytic conditions (D_2O, pD = 7.0, 25°C, no substrate) revealed no change of the ^{31}P NMR singlet peak at 2.73 ppm after one week of incubation thereby demonstrating the polyanion's pre-catalytic stability (**Fig. S11 B**), which is additionally strongly supported by the successful crystallographic characterization of **{$Y_3(HPO_3)Sb_3W_{28}$}** that can be isolated as single crystals after the aging experiments (**Tables S12, S13**).

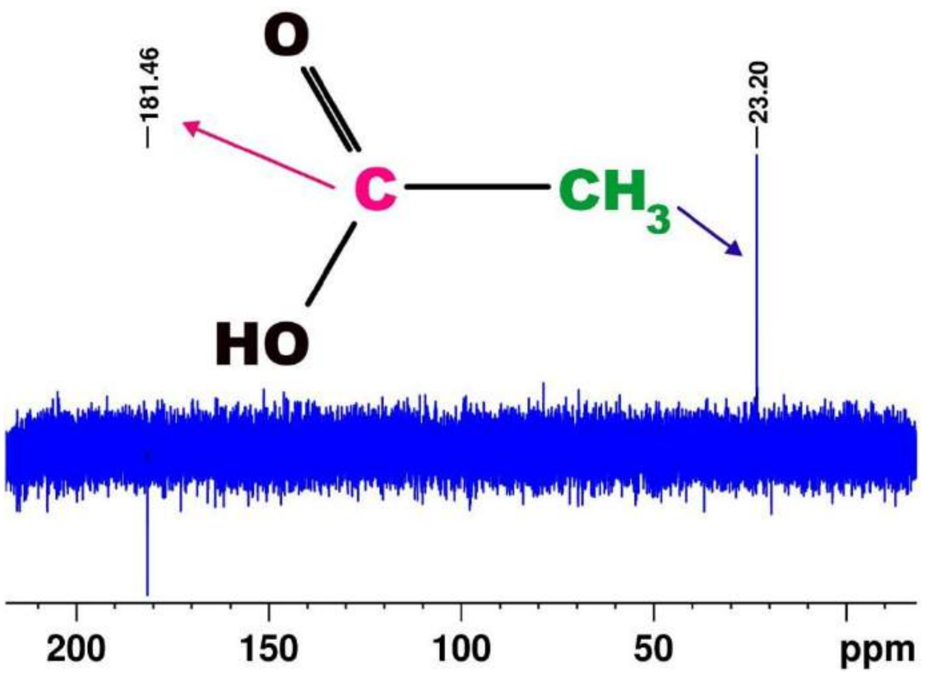

Figure S9. ^{13}C NMR spectrum of **{$Y_3(HPO_3)Sb_3W_{28}$}** (D_2O, pD = 7.0 via NaOH) revealing two peaks that can be attributed to the presence of methyl - (δ = 23.20 ppm) and carboxyl group (δ = 181.46 ppm), respectively.

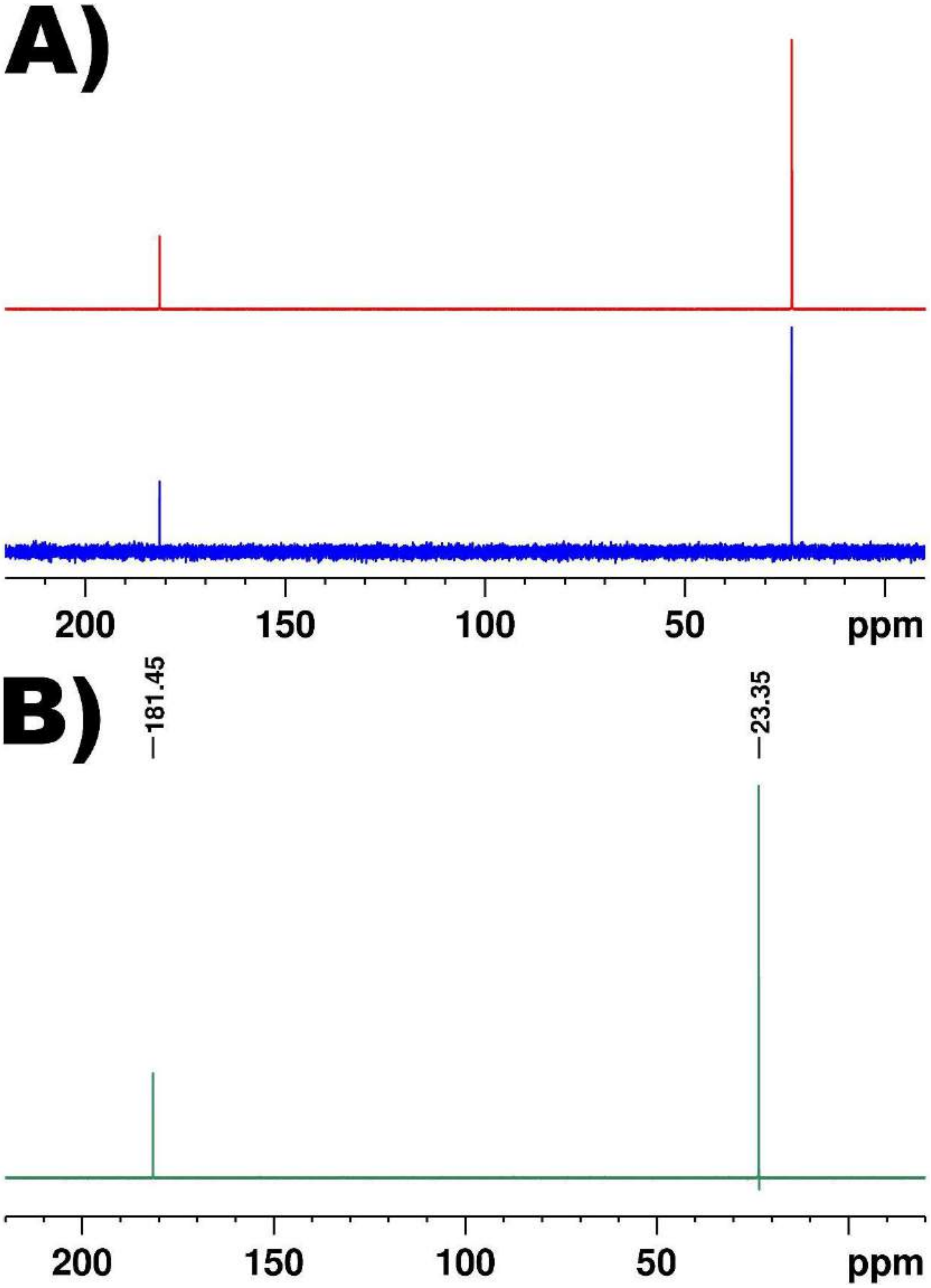

Figure S10. ^{13}C NMR spectra of **{$Y_3(HPO_3)Sb_3W_{28}$}** (D_2O, pD = 7.0 via NaOH) **A)** immediately after dissolution (blue) and after subsequent addition of NaOAc (red) leading to a substantial increase in the two peaks arising from the methyl - (δ = 23.20 ppm) and carboxyl group (δ = 181.46 ppm), respectively. **B)** The difference spectrum (green) that is obtained by subtracting the spectrum before (blue) from the spectrum after addition of NaOAc (red) shows no additional peaks demonstrating the exclusive presence of unbound acetate in solution.

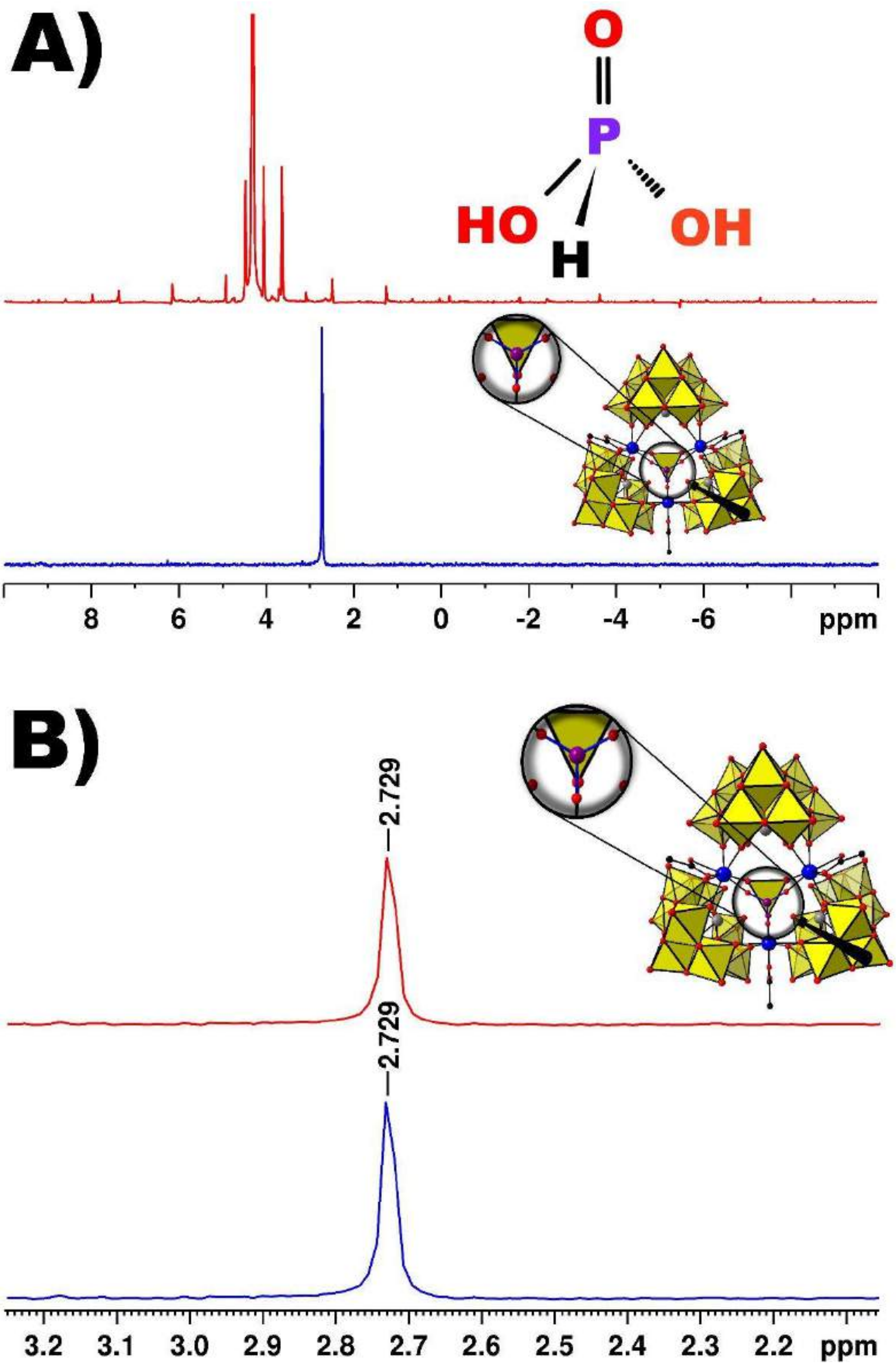

Figure S11. ^{31}P-NMR spectra comparing aqueous D_2O solutions (pD = 7.0 via DCl) of **A)** free PO_3^{3-} added as H_3PO_3 (red) and **{$Y_3(HPO_3)Sb_3W_{28}$}** (blue) and **B) {$Y_3(HPO_3)Sb_3W_{28}$}** immediately after the polyanion's dissolution (blue) and after one week of aging (25°C, red). The unchanged position of the P-NMR peak at ~2.73 ppm arising from the PO_3 motif in **{$Y_3(HPO_3)Sb_3W_{28}$}** indicates precatalytic stability of the POM catalyst.

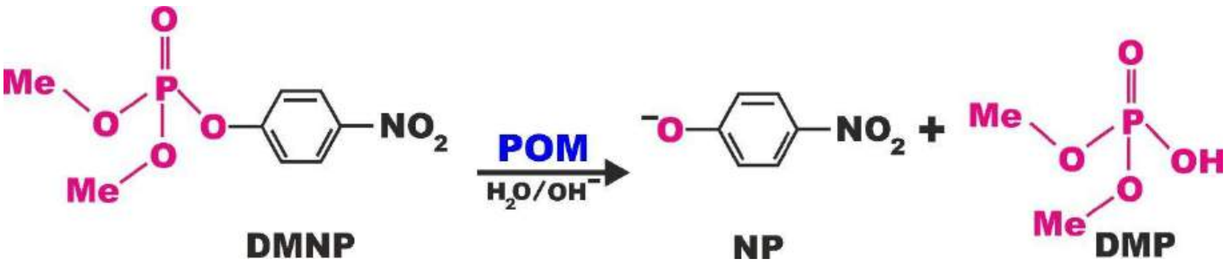

Scheme S1. Schematic representation of the POM-catalyzed hydrolysis of *O,O*-dimethyl *O*-(4-nitrophenyl) phosphate (DMNP) to p – nitrophenolate (NP) and dimethyl phosphate (DMP). Me = -CH_3.

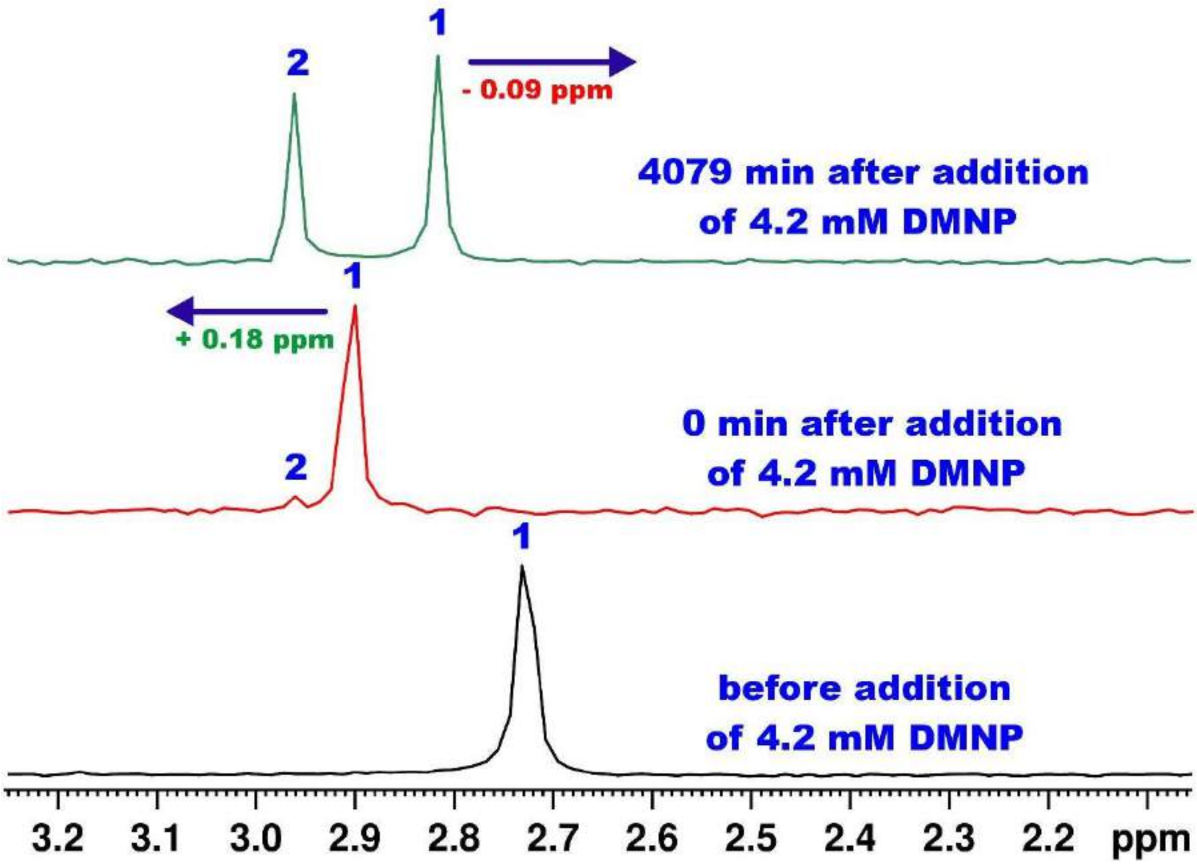

Figure S12. ^{31}P NMR spectra of **{$Y_3(HPO_3)Sb_3W_{28}$}** (**1**) exhibiting a singlet peak at 2.73 ppm before addition of DMNP. Addition of DMNP results in an immediate downfield shift of the peak to 2.91 ppm and slight formation of the hydrolysis product dimethyl phosphate (DMP) (**2**). With progressing DMNP hydrolysis, the singlet peak of **{$Y_3(HPO_3)Sb_3W_{28}$}** (**1**) undergoes an upfield shift close to its initial position at 2.73 ppm indicating stability of the polyanion under turnover conditions (Tris-HCl [125 mM], pD = 7.0 at 25°C).

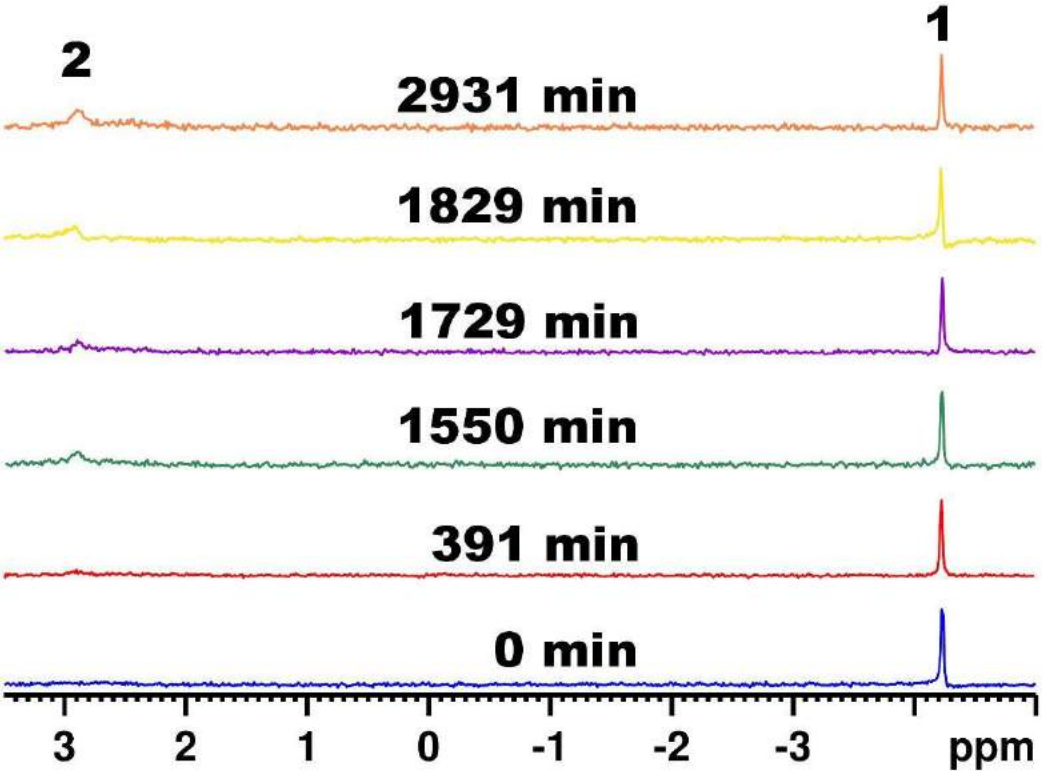

Figure S13. ^{31}P NMR spectra of the hydrolysis of DMNP (**1**) [4.2 mM] to nitrophenol (NP) and dimethyl phosphate (DMP) (**2**) with **{$Gd_3(HPO_3)Sb_3W_{28}$}** [2.5 mM] in Tris-HCl [125 mM] at pD 7.0 25 °C.

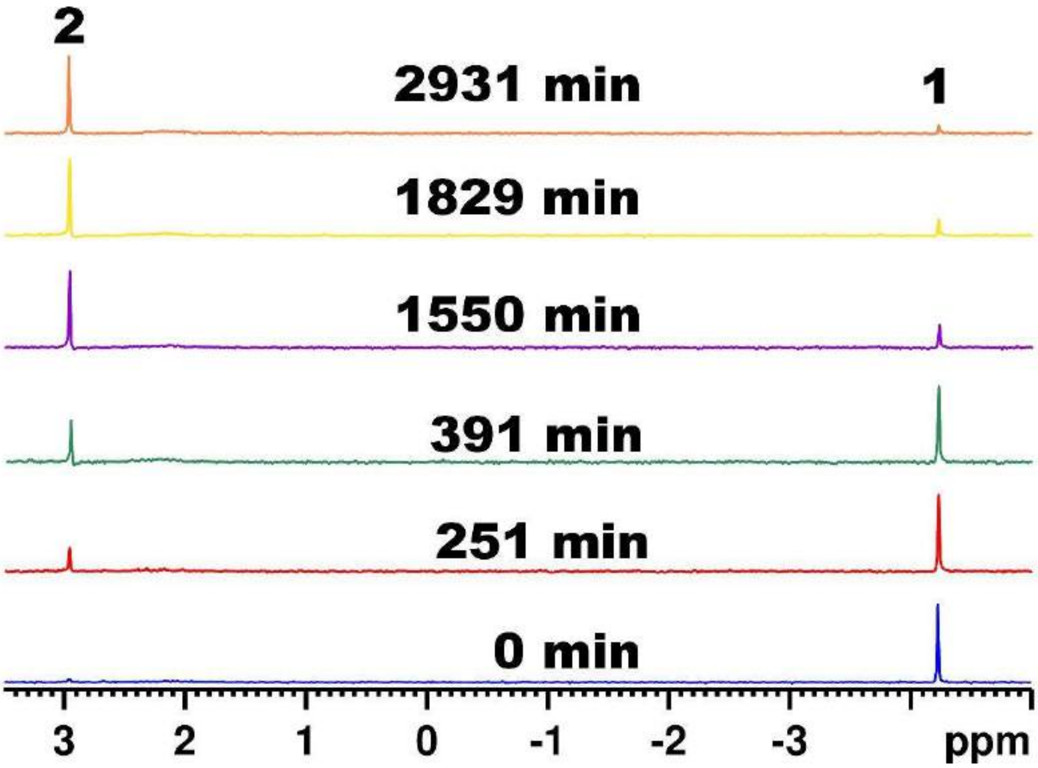

Figure S14. ^{31}P NMR spectra of the hydrolysis of DMNP (**1**) [4.2 mM] to nitrophenol (NP) and dimethyl phosphate (DMP) (**2**) with **{$Yb_3(HPO_3)Sb_3W_{28}$}** [2.5 mM] in Tris-HCl [125 mM] at pD 7.0 25 °C.

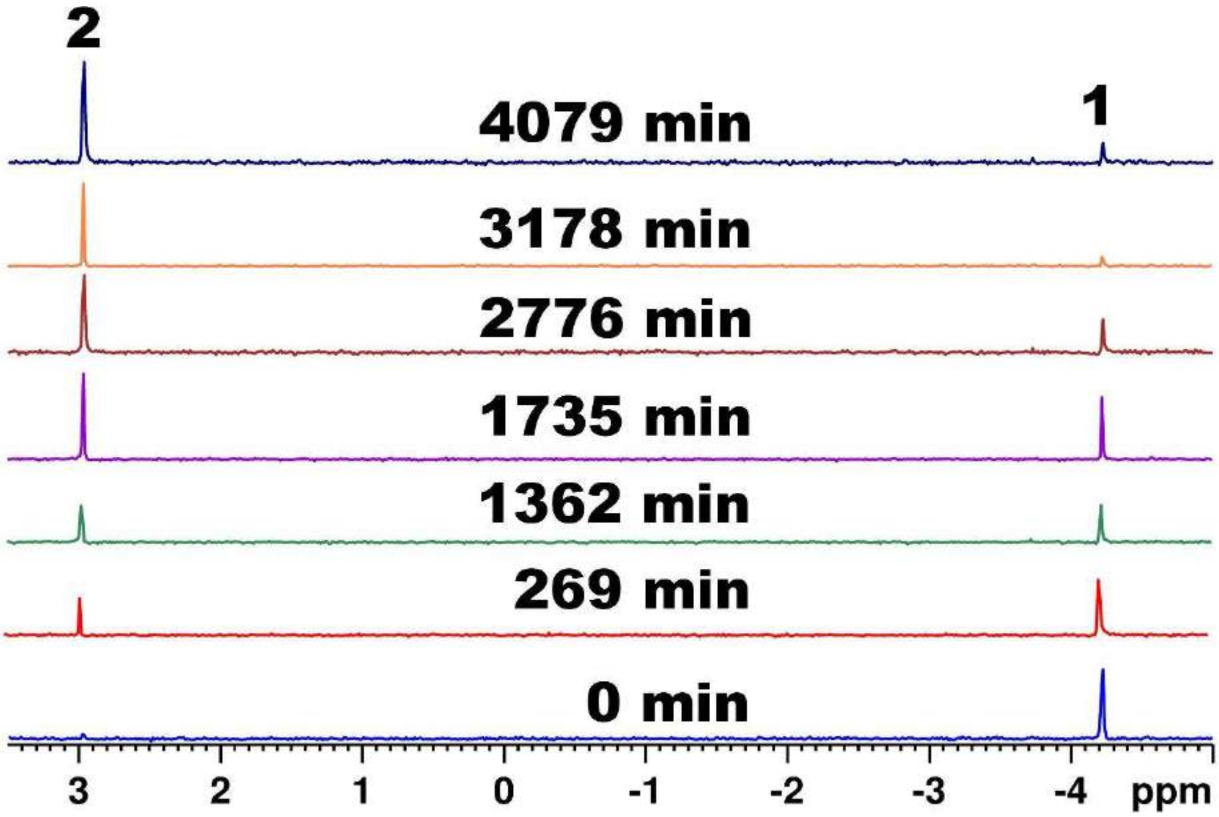

Figure S15. ^{31}P NMR spectra of the hydrolysis of DMNP (**1**) [4.2 mM] to nitrophenol (NP) and dimethyl phosphate (DMP) (**2**) with **{SbW$_9$}** [2.5 mM] in Tris-HCl [125 mM] at pD 7.0 25 °C.

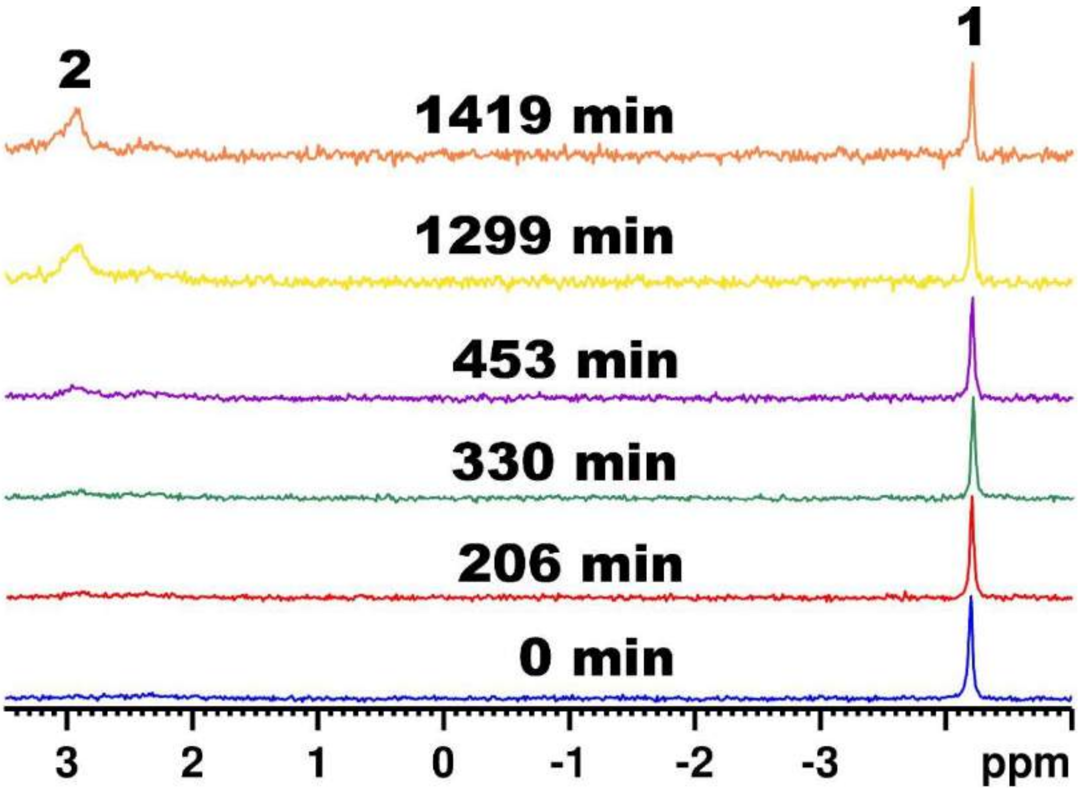

Figure S16. ^{31}P NMR spectra of the hydrolysis of DMNP (**1**) [4.2 mM] to nitrophenol (NP) and dimethyl phosphate (DMP) (**2**) with **{$Gd_3(HPO_3)Sb_3W_{28}$}** [2.5 mM] in D_2O at pD 7.0 via NaOD, 25 °C.

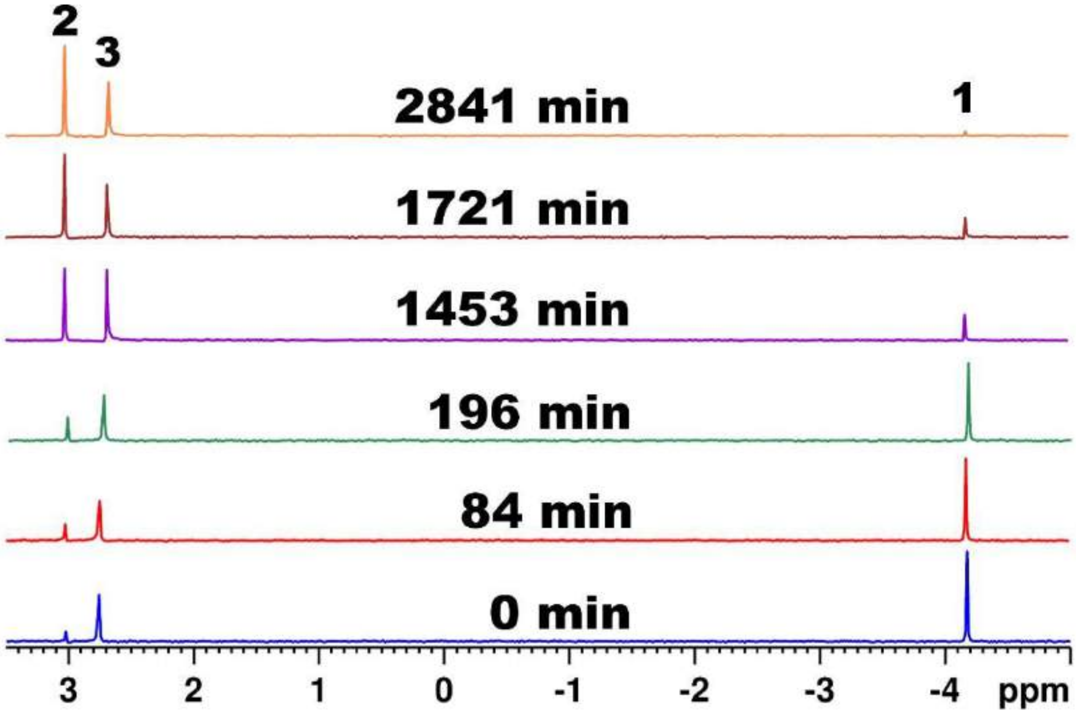

Figure S17. ^{31}P NMR spectra of the hydrolysis of DMNP (**1**) [4.2 mM] to nitrophenol (NP) and dimethyl phosphate (DMP) (**2**) with **{$Y_3(HPO_3)Sb_3W_{28}$}** [2.5 mM]] in D_2O at pD 7.0 *via* NaOD, 25 °C. The virtually unchanged singlet peak at 2.81 ppm corresponds to the {HPO_3} group (**3**) incorporated in the diamagnetic representative **{$Y_3(HPO_3)Sb_3W_{28}$}** suggesting solution stability of the isostructural series under turnover conditions.

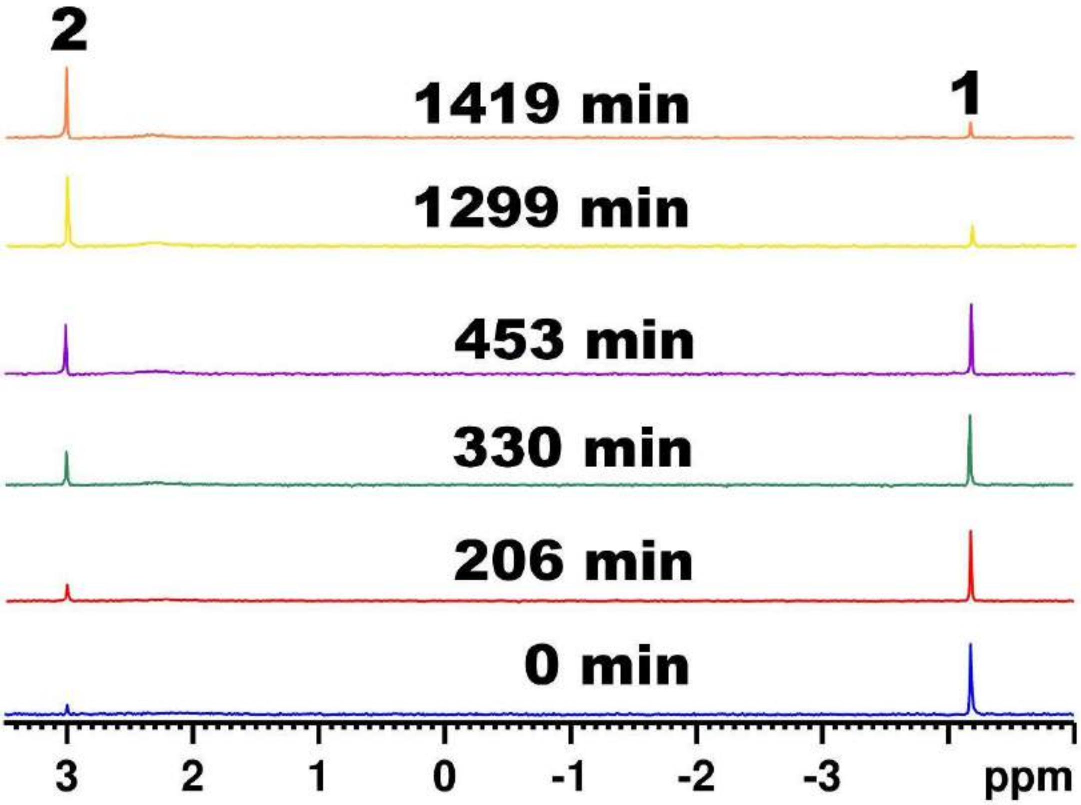

Figure S18. ^{31}P NMR spectra of the hydrolysis of DMNP (**1**) [4.2 mM] to nitrophenol (NP) and dimethyl phosphate (DMP) (**2**) with **{$Yb_3(HPO_3)Sb_3W_{28}$}** [2.5 mM] in D_2O at pD 7.0 via NaOD, 25 °C.

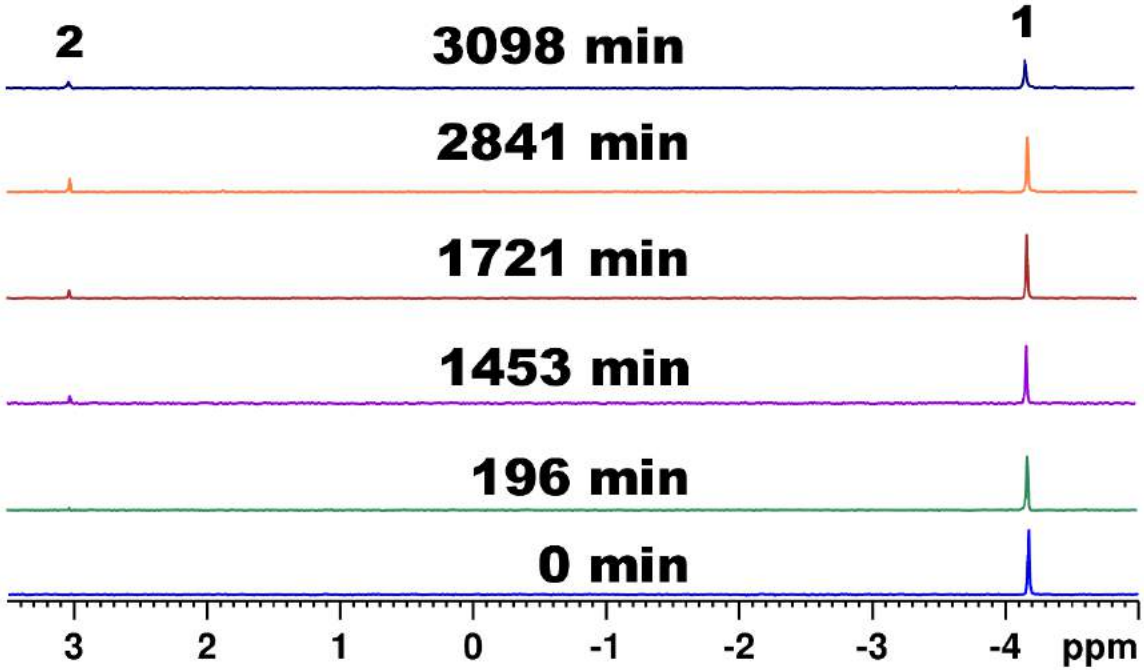

Figure S19. ^{31}P NMR spectra of the hydrolysis of DMNP (**1**) [4.2 mM] to nitrophenol (NP) and dimethyl phosphate (DMP) (**2**) with **{SbW_9}** [2.5 mM] in D_2O at pD 7.0 via NaOD, 25 °C.

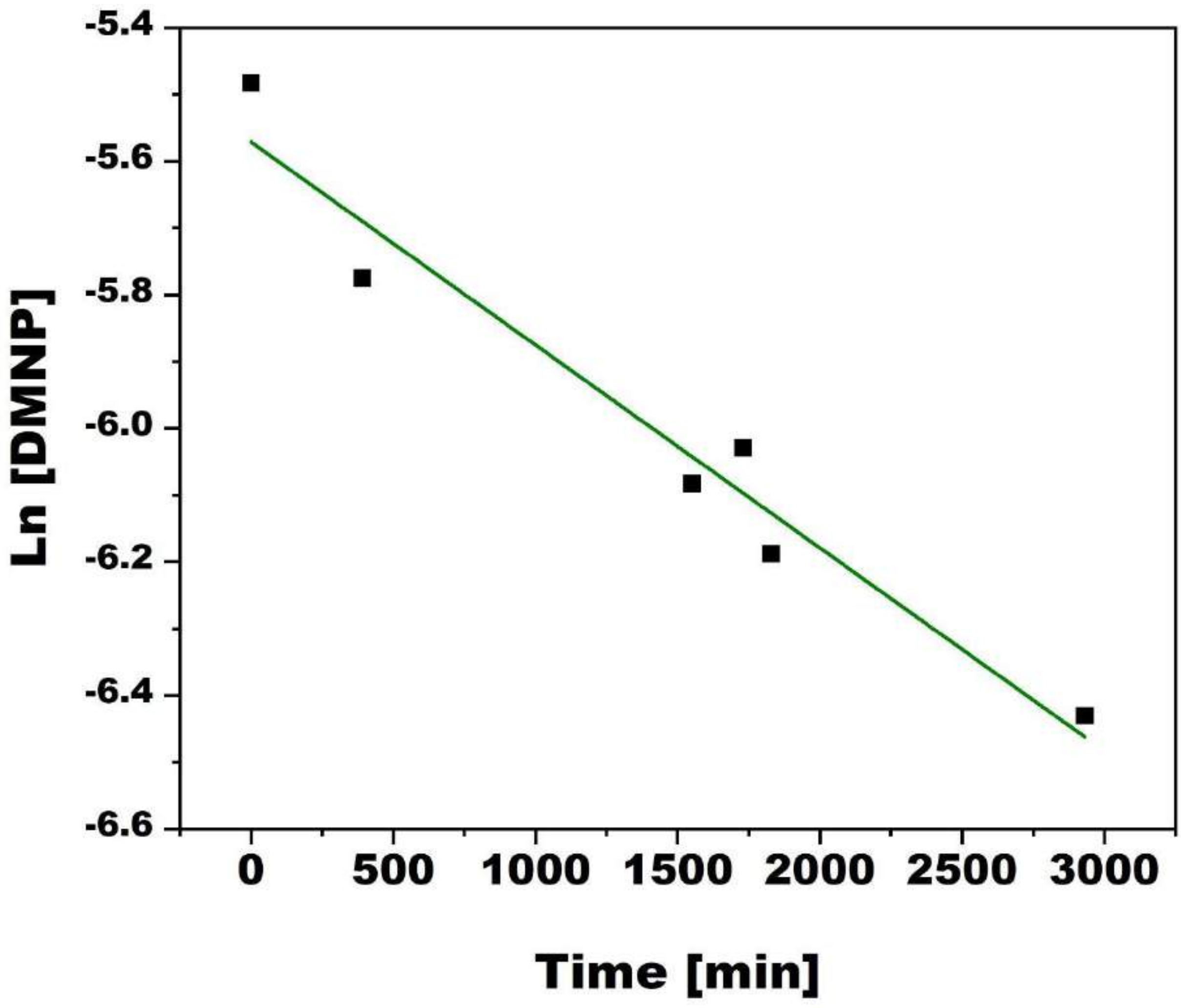

Figure S20. Decrease of *O,O*-dimethyl *O*-(4-nitrophenyl) phosphate (**DMNP**) concentration as a function of time using the logarithmic *O,O*-dimethyl *O*-(4-nitrophenyl) phosphate (**DMNP**) concentration values obtained from the NMR integration values after reaction with **{$Gd_3(HPO_3)Sb_3W_{28}$}** in Tris-HCl [125 mM], pH = 7.0, room temperature (25°C) for 0, 391, 1550, 1729, 1829, and 2931 min. The function is described by the linear fit (R^2 = 0.95): ln ([DMNP]) = -3.04 (± 0.34) × 10^{-4} x – 5.57 with x being the reaction time in min. The slope of the linear fit gives the rate constant k = 3.04 (± 0.34) × 10^{-4} min^{-1}.

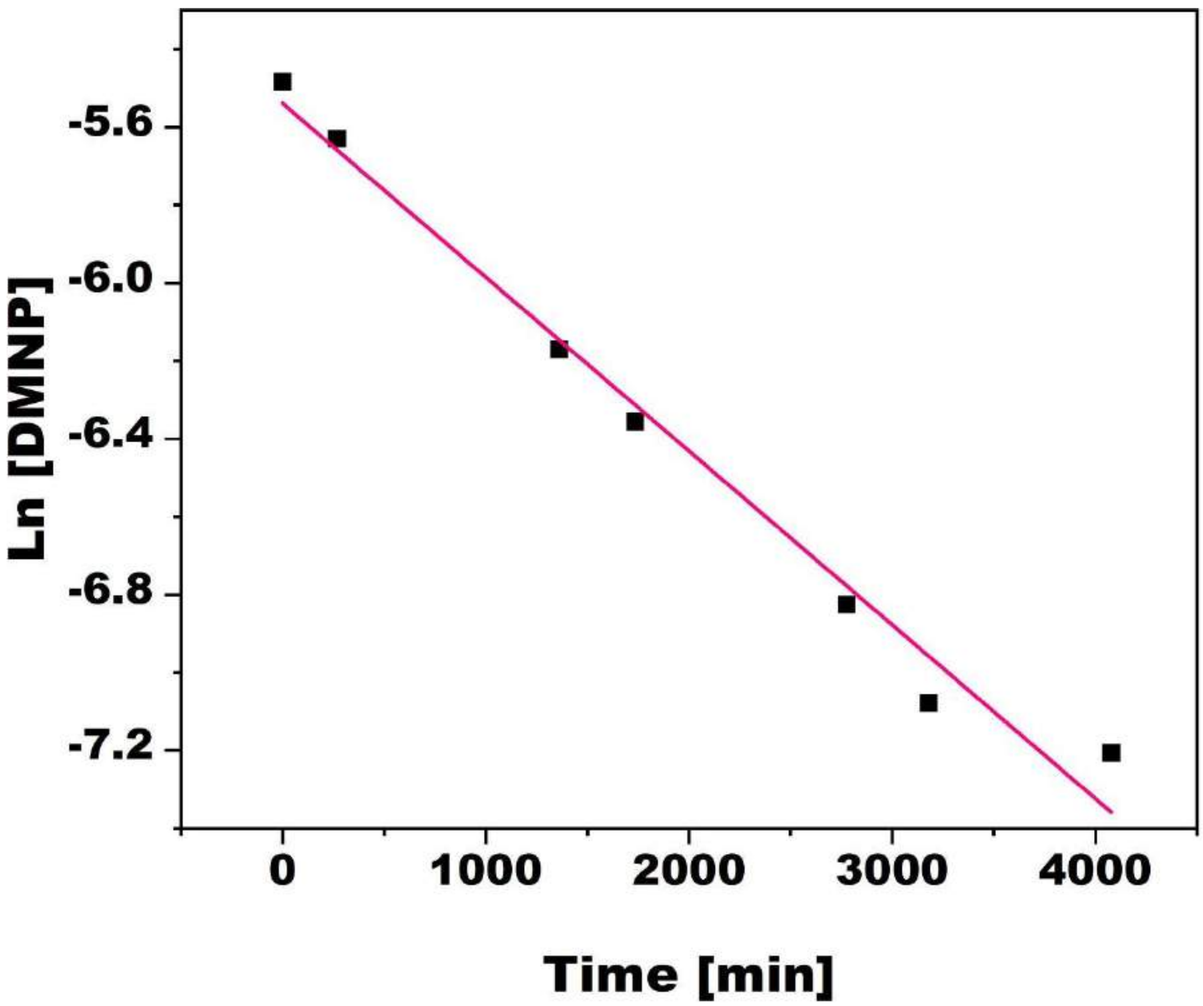

Figure S21. Decrease of *O,O*-dimethyl *O*-(4-nitrophenyl) phosphate (**DMNP**) concentration as a function of time using the logarithmic *O,O*-dimethyl *O*-(4-nitrophenyl) phosphate (**DMNP**) concentration values obtained from the NMR integration values after reaction with **{$Y_3(HPO_3)Sb_3W_{28}$}** in Tris-HCl [125 mM], pH = 7.0, room temperature (25°C) for 0, 269, 1362, 1735, 2776, 3178, and 4079 min. The function is described by the linear fit (R^2 = 0.98): ln ([DMNP]) = -4.46 (± 0.26) × 10^{-4} x – 6.06 with x being the reaction time in min. The slope of the linear fit gives the rate constant k = 4.46 (± 0.26) × 10^{-4} min^{-1}.

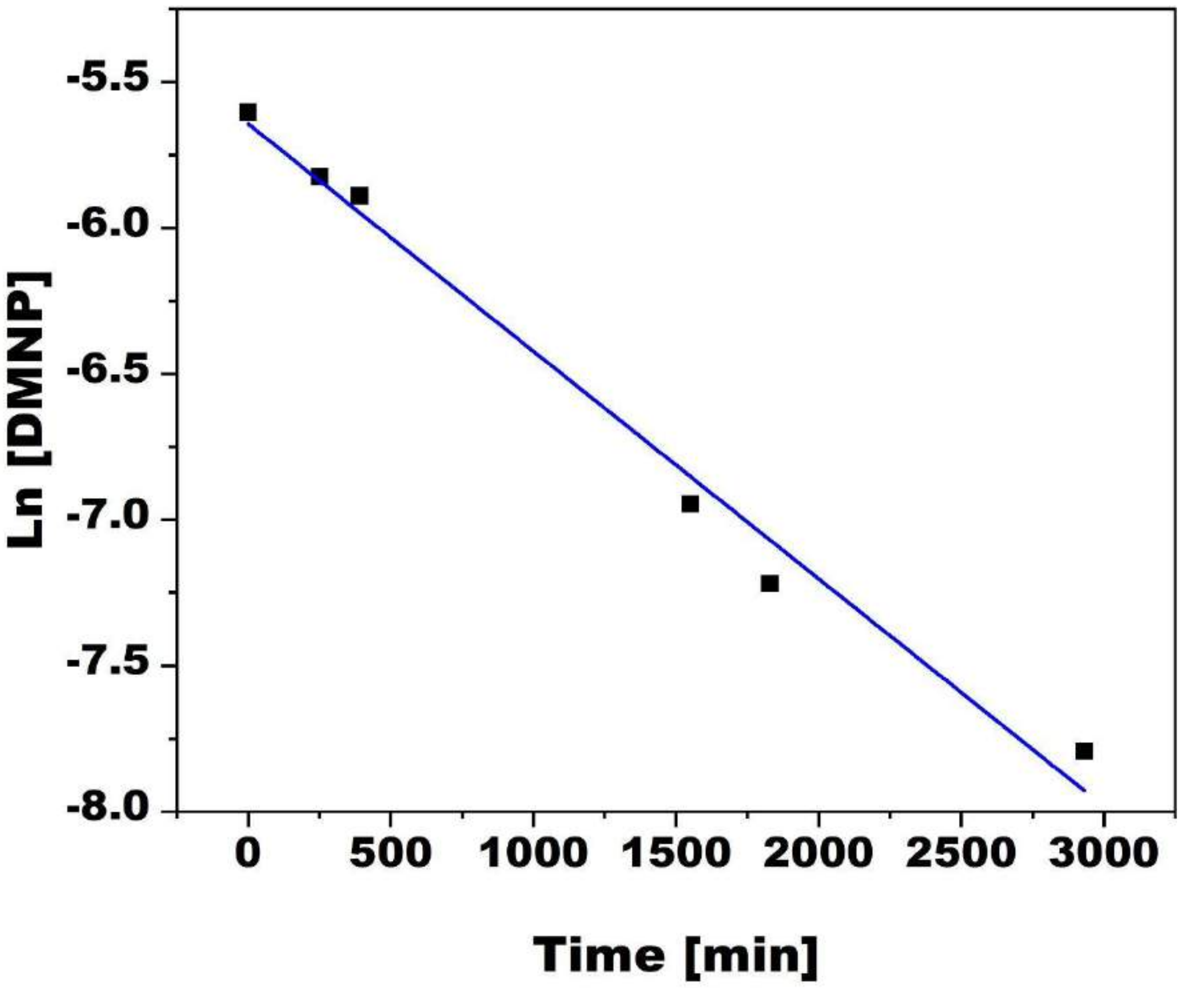

Figure S22. Decrease of *O,O*-dimethyl *O*-(4-nitrophenyl) phosphate (**DMNP**) concentration as a function of time using the logarithmic *O,O*-dimethyl *O*-(4-nitrophenyl) phosphate (**DMNP**) concentration values obtained from the NMR integration values after reaction with **{$Yb_3(HPO_3)Sb_3W_{28}$}** in Tris-HCl [125 mM], pH = 7.0, room temperature (25°C) for 0, 251, 391, 1550, 1829, and 2931 min. The function is described by the linear fit (R^2 = 0.99): ln ([DMNP]) = -7.79 (± 0.46) × 10^{-4} x – 5.64 with x being the reaction time in min. The slope of the linear fit gives the rate constant k = 7.79 (± 0.46) × 10^{-4} min^{-1}.

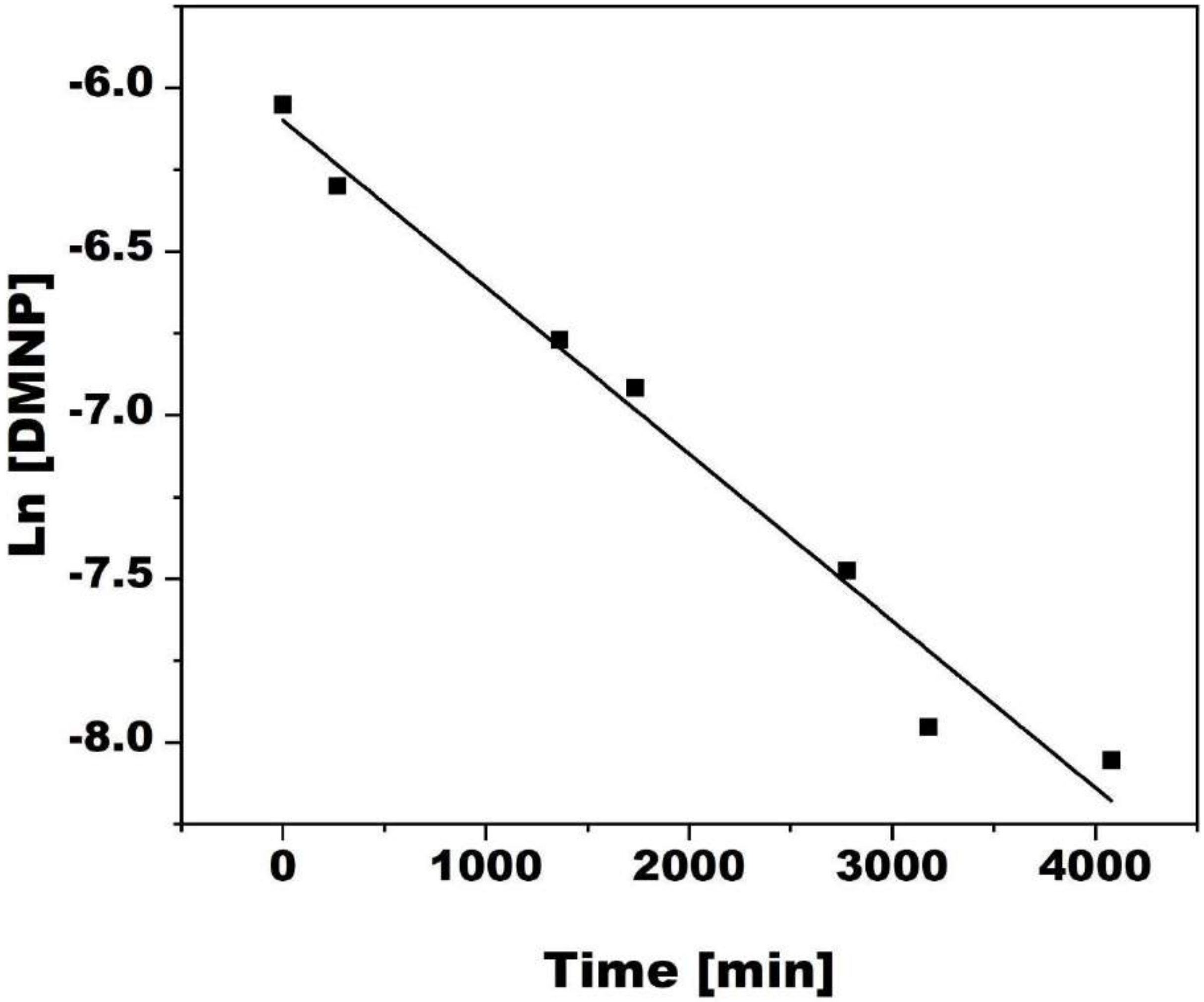

Figure S23. Decrease of *O,O*-dimethyl *O*-(4-nitrophenyl) phosphate (**DMNP**) concentration as a function of time using the logarithmic *O,O*-dimethyl *O*-(4-nitrophenyl) phosphate (**DMNP**) concentration values obtained from the NMR integration values after reaction with **{SbW$_9$}** in Tris-HCl [125 mM], pH = 7.0, room temperature (25°C) for 0, 269, 1362, 1735, 2776, 3178, and 4079 min. The function is described by the linear fit (R^2 = 0.98): ln ([DMNP]) = -5.10 (±0.35) × 10^{-4} x – 6.10 with x being the reaction time in min. The slope of the linear fit gives the rate constant k = 5.10 (± 0.35) × 10^{-4} min^{-1}.

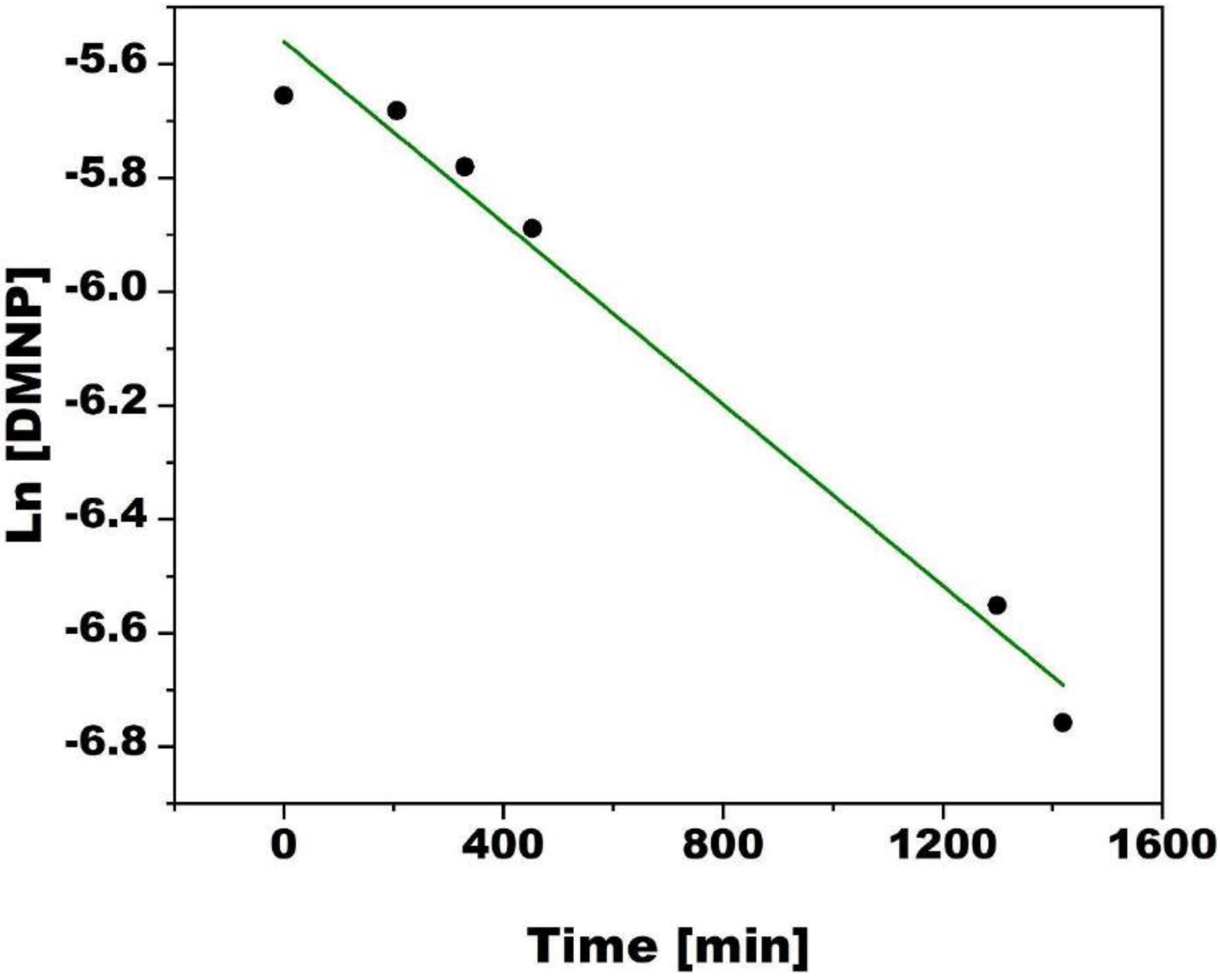

Figure S24. Decrease of *O,O*-dimethyl *O*-(4-nitrophenyl) phosphate (**DMNP**) concentration as a function of time using the logarithmic *O,O*-dimethyl *O*-(4-nitrophenyl) phosphate (**DMNP**) concentration values obtained from the NMR integration values after reaction with **{$Gd_3(HPO_3)Sb_3W_{28}$}** in Tris-HCl [125 mM], pH = 7.0, room temperature (25°C) for 0, 206, 330, 453, 1299, and 1419 min. The function is described by the linear fit ($R^2 = 0.98$): ln ([DMNP]) = -7.97 (± 0.53) × 10^{-4} x – 5.56 with x being the reaction time in min. The slope of the linear fit gives the rate constant k = 7.97 (± 0.53) × 10^{-4} min^{-1}.

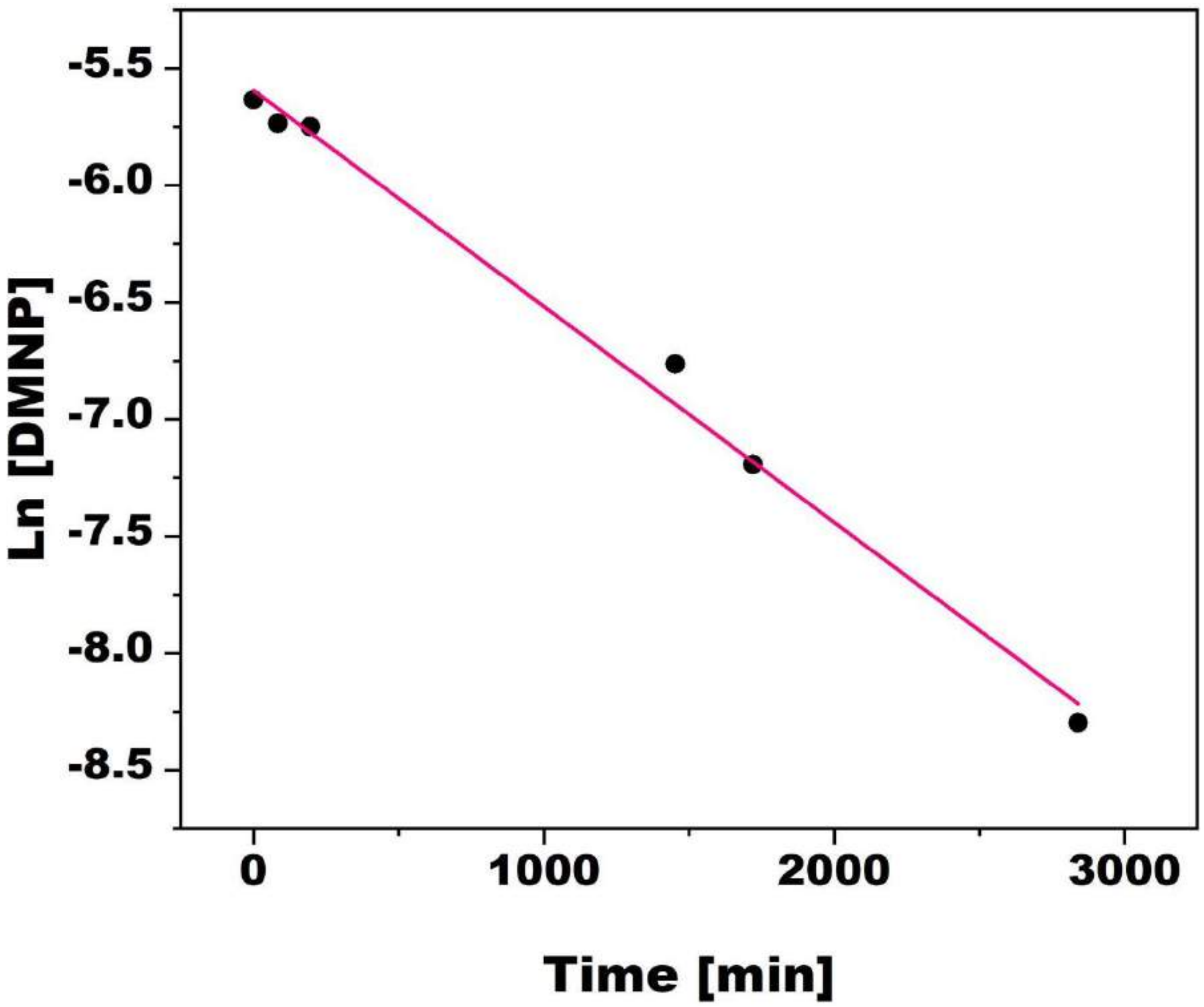

Figure S25. Decrease of *O,O*-dimethyl *O*-(4-nitrophenyl) phosphate (**DMNP**) concentration as a function of time using the logarithmic *O,O*-dimethyl *O*-(4-nitrophenyl) phosphate (**DMNP**) concentration values obtained from the NMR integration values after reaction with **{$Y_3(HPO_3)Sb_3W_{28}$}** in D_2O (pD = 7.0 via NaOD), room temperature (25°C) for 0, 84, 196, 1453, 1721, and 2841 min. The function is described by the linear fit (R^2 = 0.99): ln ([DMNP]) = -9.23 (± 0.40) × 10^{-4} x – 5.59 with x being the reaction time in min. The slope of the linear fit gives the rate constant k = 9.23 (± 0.40) × 10^{-4} min^{-1}.

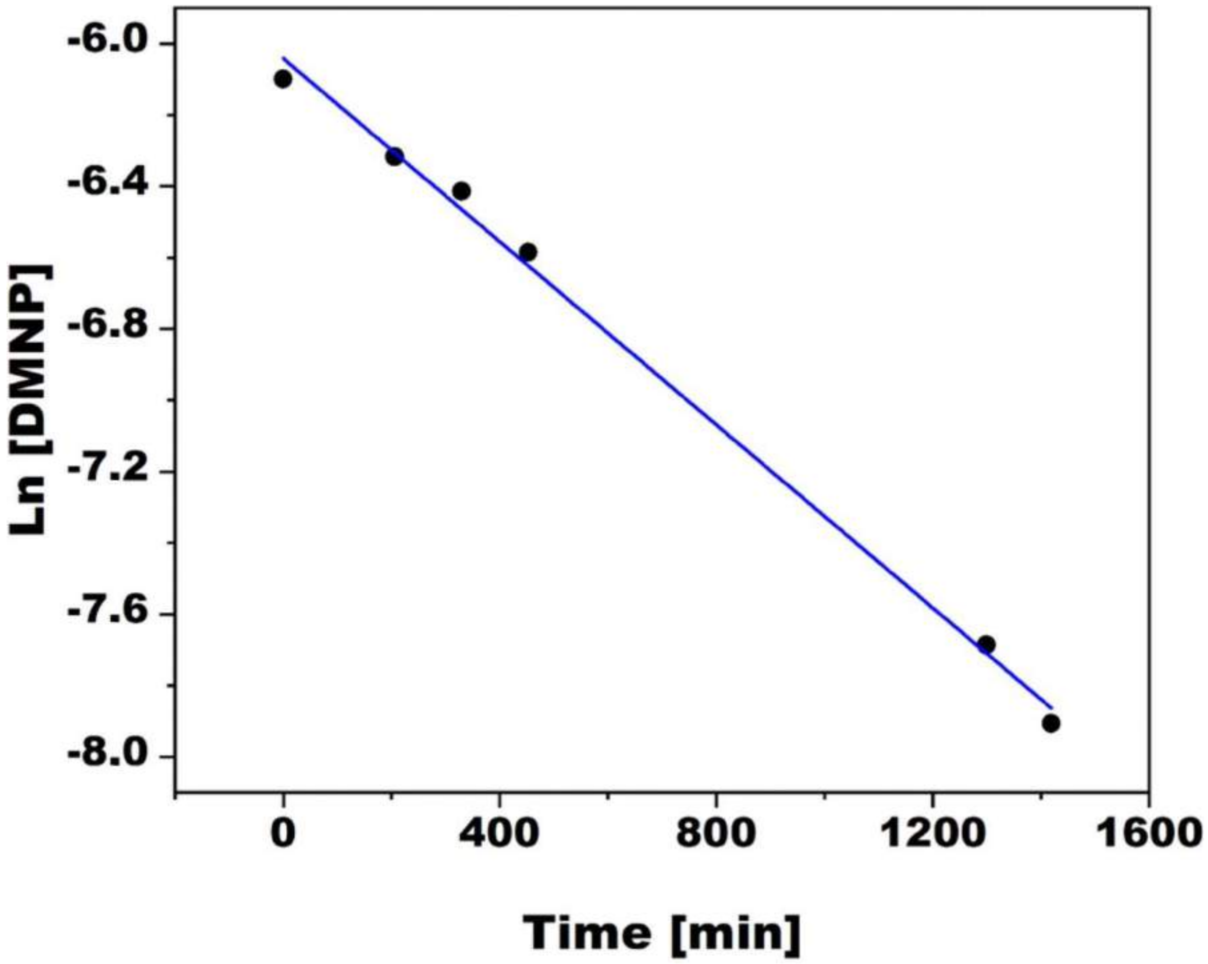

Figure S26. Decrease of *O,O*-dimethyl *O*-(4-nitrophenyl) phosphate (**DMNP**) concentration as a function of time using the logarithmic *O,O*-dimethyl *O*-(4-nitrophenyl) phosphate (**DMNP**) concentration values obtained from the NMR integration values after reaction with **{$Yb_3(HPO_3)Sb_3W_{28}$}** in D_2O [125 mM], pH = 7.0, room temperature (25°C) for 0, 206, 330, 453, 1299, and 1419 min. The function is described by the linear fit (R^2 = 0.99): ln ([DMNP]) = - 12.79 (± 0.38) × 10^{-4} x – 6.04 with x being the reaction time in min. The slope of the linear fit gives the rate constant *k* = 12.79 (± 0.38) × 10^{-4} min^{-1}.

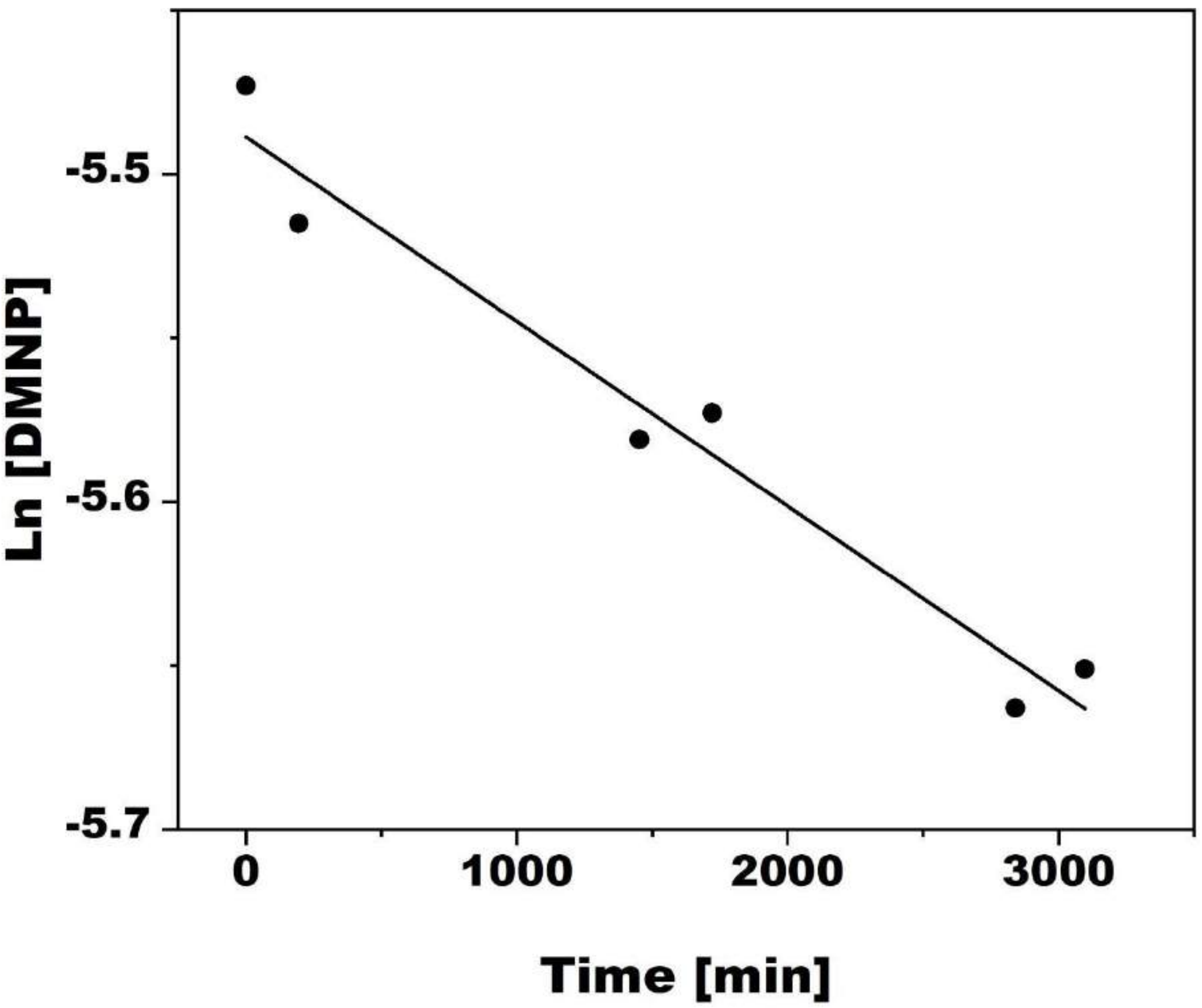

Figure S27. Decrease of *O,O*-dimethyl *O*-(4-nitrophenyl) phosphate (**DMNP**) concentration as a function of time using the logarithmic *O,O*-dimethyl *O*-(4-nitrophenyl) phosphate (**DMNP**) concentration values obtained from the NMR integration values after reaction with **{SbW_9}** in D_2O (pD = 7.0 via NaOD), room temperature (25°C) for 0, 196, 1453, 1721, 2841 and 3098 min. The function is described by the linear fit ($R^2 = 0.97$): ln ([DMNP]) = -5.63 (± 0.57) × 10^{-5} x – 5.49 with x being the reaction time in min. The slope of the linear fit gives the rate constant k = 5.63 (± 0.57) × 10^{-5} min^{-1}.

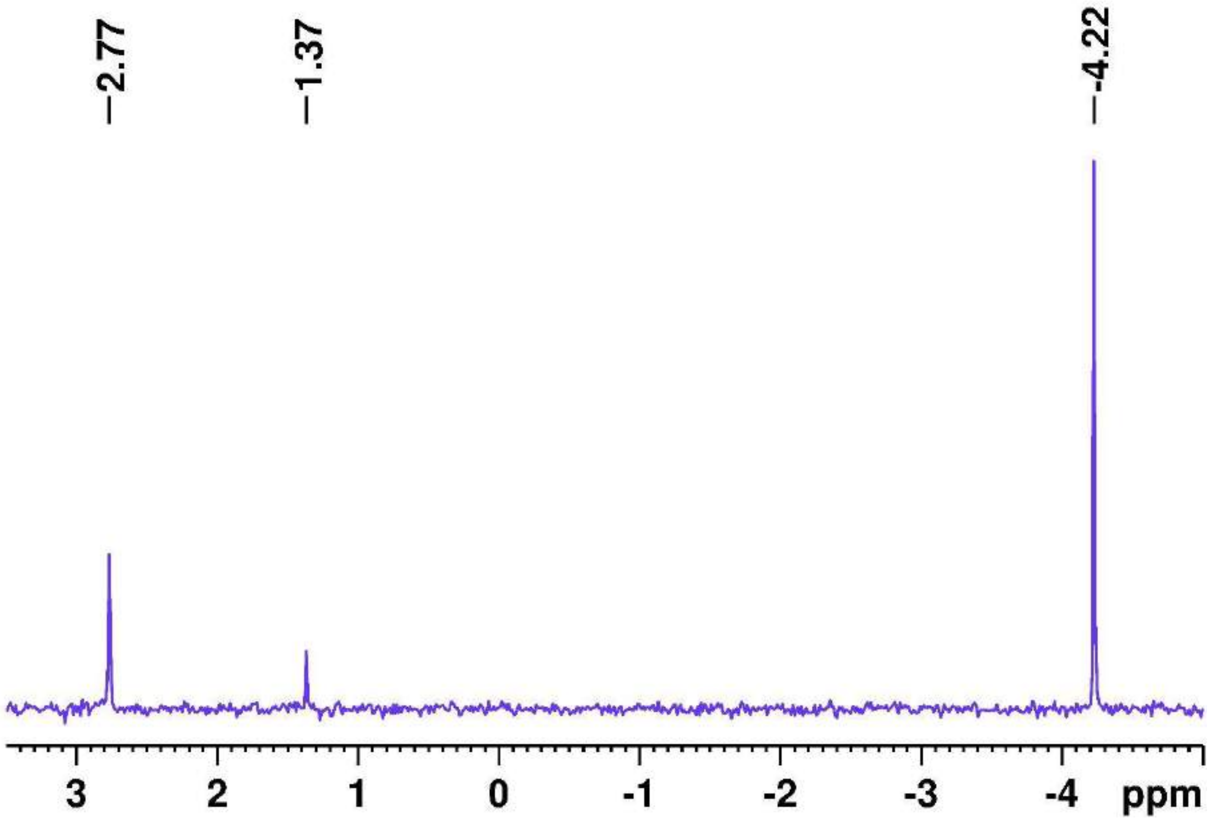

Figure S28. ^{31}P NMR spectrum recorded immediately after addition of 2.5 mM of YCl_3 to a solution containing 4.2 mM of the substrate DMNP (Tris-HCl, [125 mM] at 25°C). Aside from the hydrolysis product DMP (2.77 ppm), a clear additional peak at 1.37 ppm occurs along with slight formation of precipitates, which suggests unspecific formation of unidentifiable Y-complexes as a result of the Lewis-acid metal center’s unshielded nature.

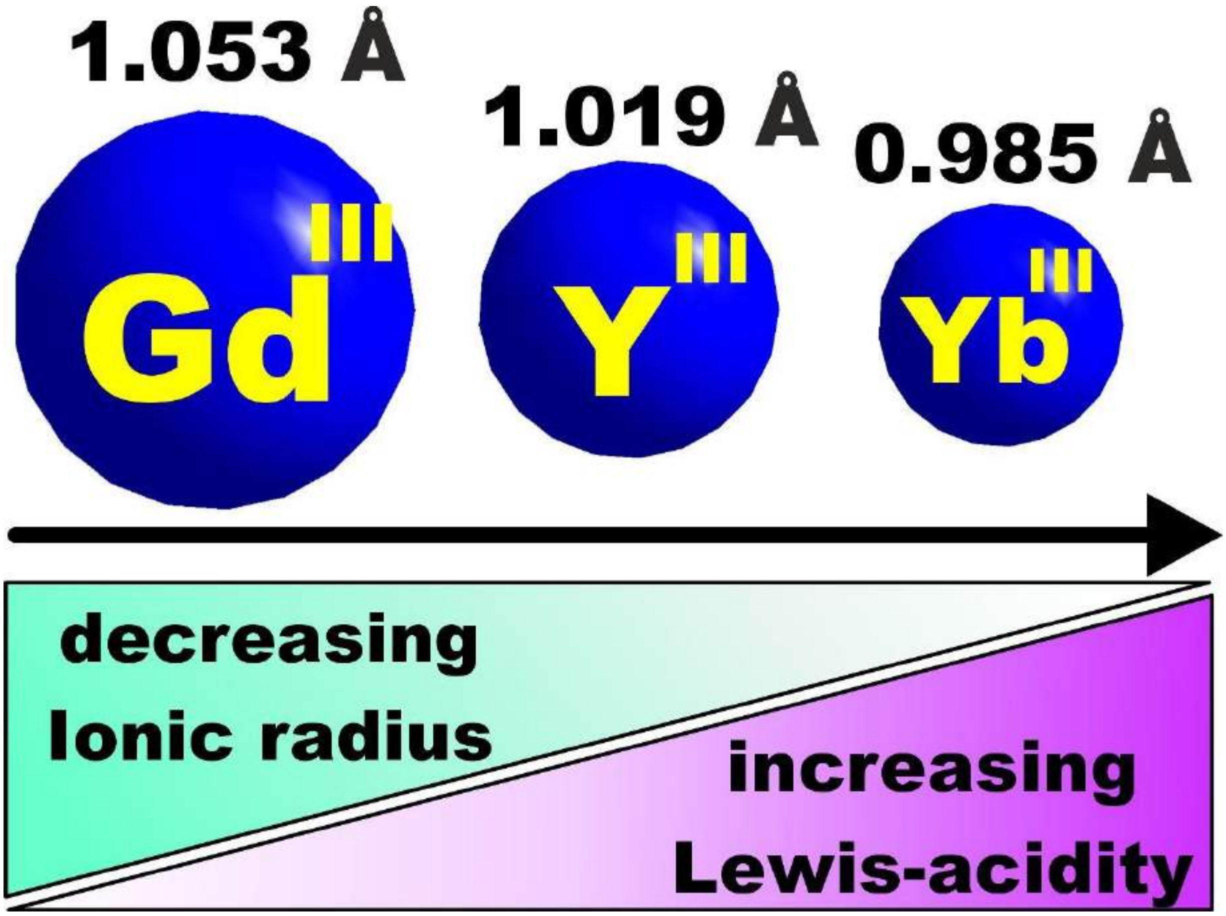

Figure S29. Schematic representation of the increasing Lewis-acid catalytic activity observed for the representatives of the **{$M_3(HPO_3)Sb_3W_{28}$}** (M = Gd^{III}, Y^{III}, and Yb^{III}) series as a result of the decreasing ionic radius. The values of the depicted ionic radii are based on the studies by Shannon.[10]

8.2. Post-catalytic stability studies

The post-catalytic stability of (POM)-catalysts in homogeneous systems is a frequently encountered issue since the use of NMR-spectroscopic techniques under turnover conditions as a powerful method to prove solution stability is mostly hampered by the low solubility and sensitivity of the ^{183}W nucleus (14.3 % natural abundance), strong paramagnetic interactions within the polyanion, or the lack of “NMR-active” nuclei with a suitable multiplicity and abundance. Attributed to the presence of the $\{HPO_3\}$-motif in all **TA**-catalysts and the diamagnetic nature of Y(III), the polyanions’ speciation behavior under turnover conditions could be monitored employing ^{31}P NMR spectroscopy on catalytic solutions containing **$\{Y_3(HPO_3)Sb_3W_{28}\}$** as a representative member of the isostructural **TA** compound series. Upon addition of DMNP to a solution containing **$\{Y_3(HPO_3)Sb_3W_{28}\}$**, an immediate downfield - shift of the singlet from 2.73 ppm to 2.91 ppm can be observed indicating the substrate’s coordination to the POM-catalyst (**Fig. S12**). Over the course of the reaction, the singlet exhibits an upfield - shift to 2.82 ppm close to its initial position without the occurrence of any additional peaks directly demonstrating the post-catalytic stability of the **$\{M_3(HPO_3)Sb_3W_{28}\}$** catalyst (**Fig. S12**, **S34A**), which is additionally supported by post-catalytic IR-spectroscopic measurements and energy dispersive X-ray analysis (EDX) that were conducted on the precipitated cesium-salts of the **$\{M_3(HPO_3)Sb_3W_{28}\}$** polyanions, thereby clearly showing the characteristic W-O-W bridging and terminal W=O vibrations in the tungsten fingerprint area from 300 – 1000 cm^{-1} (**Fig. S30 – S32**) and a comparable Sb:W ratio between the pre-catalytic **$\{Y_3(HPO_3)Sb_3W_{28}\}$** and the post-catalytically isolated cesium salt **$Cs\{Y_3(HPO_3)Sb_3W_{28}\}$** (**Fig. S33**). Subsequent control experiments with YCl_3 as a non-shielded free Lewis-metal center resulted in the immediate appearance of a singlet at 1.37 suggesting the formation of an unidentifiable Y-complex, even at low concentrations of the metal center, which additionally proves the solution stability of the POM compounds under reaction conditions (**Fig. S28**).

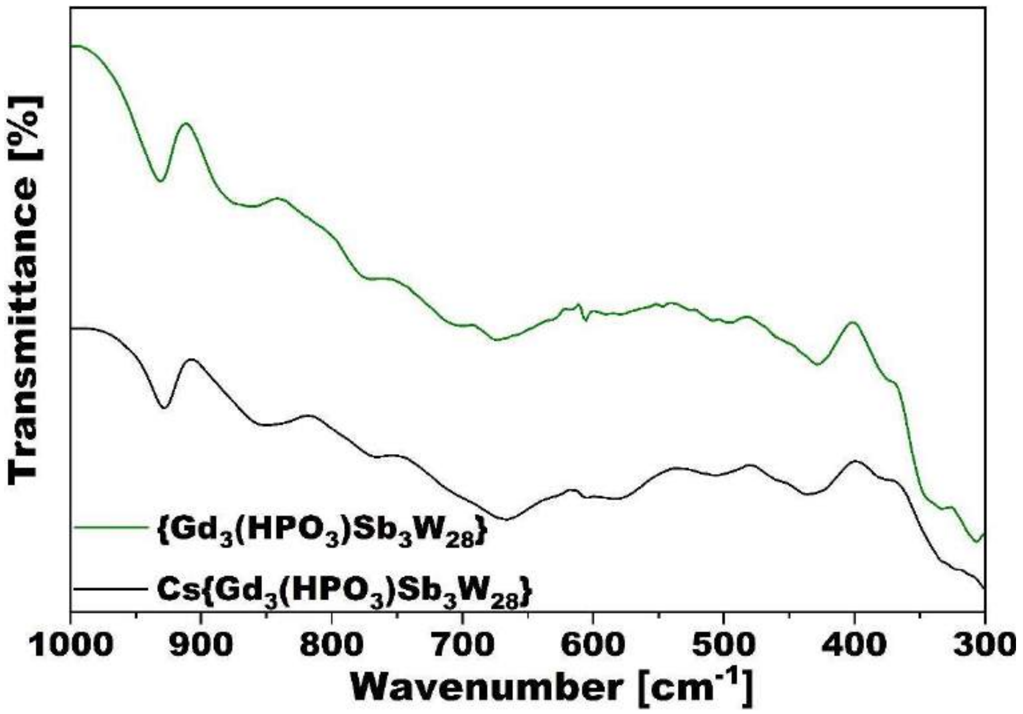

Figure S30. IR-spectra showing the tungsten fingerprint area from 300 - 1000 cm^{-1} of **{$Gd_3(HPO_3)Sb_3W_{28}$}**, (green) and the precipitated polyanion catalyst by addition of CsCl after reaction with DMNP (DMNP = *O,O*-dimethyl *O*-(4-nitrophenyl) phosphate) for 100 h at room temperature (25°C), pD = 7.0 **Cs{$Gd_3(HPO_3)Sb_3W_{28}$}** (black).

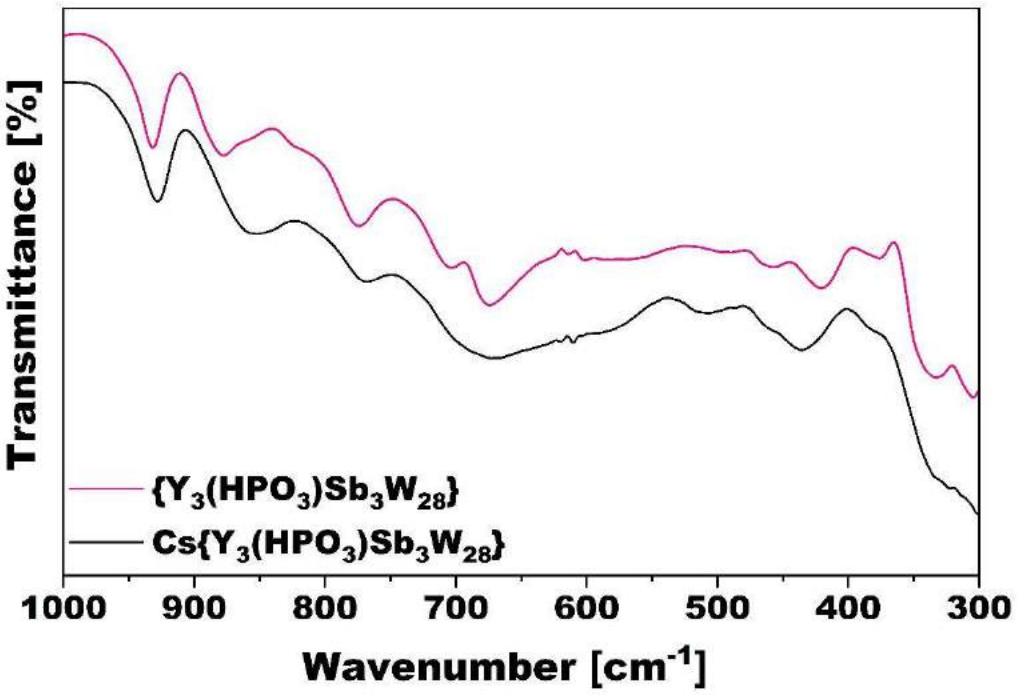

Figure S31. IR-spectra showing the tungsten fingerprint area from 300 - 1000 cm^{-1} of **{$Y_3(HPO_3)Sb_3W_{28}$}**, (pink) and the precipitated polyanion catalyst by addition of CsCl after reaction with DMNP (DMNP = *O,O*-dimethyl *O*-(4-nitrophenyl) phosphate) for 100 h at room temperature (25°C), pD = 7.0 **Cs{$Y_3(HPO_3)Sb_3W_{28}$}** (black).

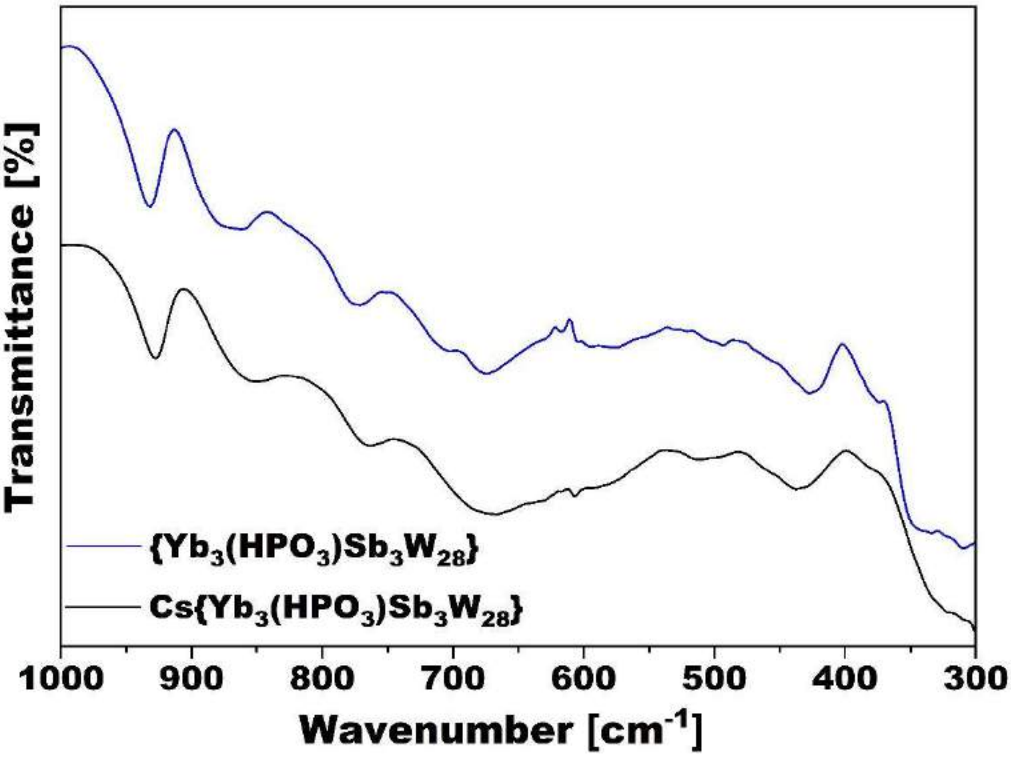

Figure S32. IR-spectra showing the tungsten fingerprint area from 300 - 1000 cm^{-1} of **{$Yb_3(HPO_3)Sb_3W_{28}$}**, (blue) and the precipitated polyanion catalyst by addition of CsCl after reaction with DMNP (DMNP = *O*,*O*-dimethyl *O*-(4-nitrophenyl) phosphate) for 100 h at room temperature (25°C), pD = 7.0 **Cs{$Yb_3(HPO_3)Sb_3W_{28}$}** (black).

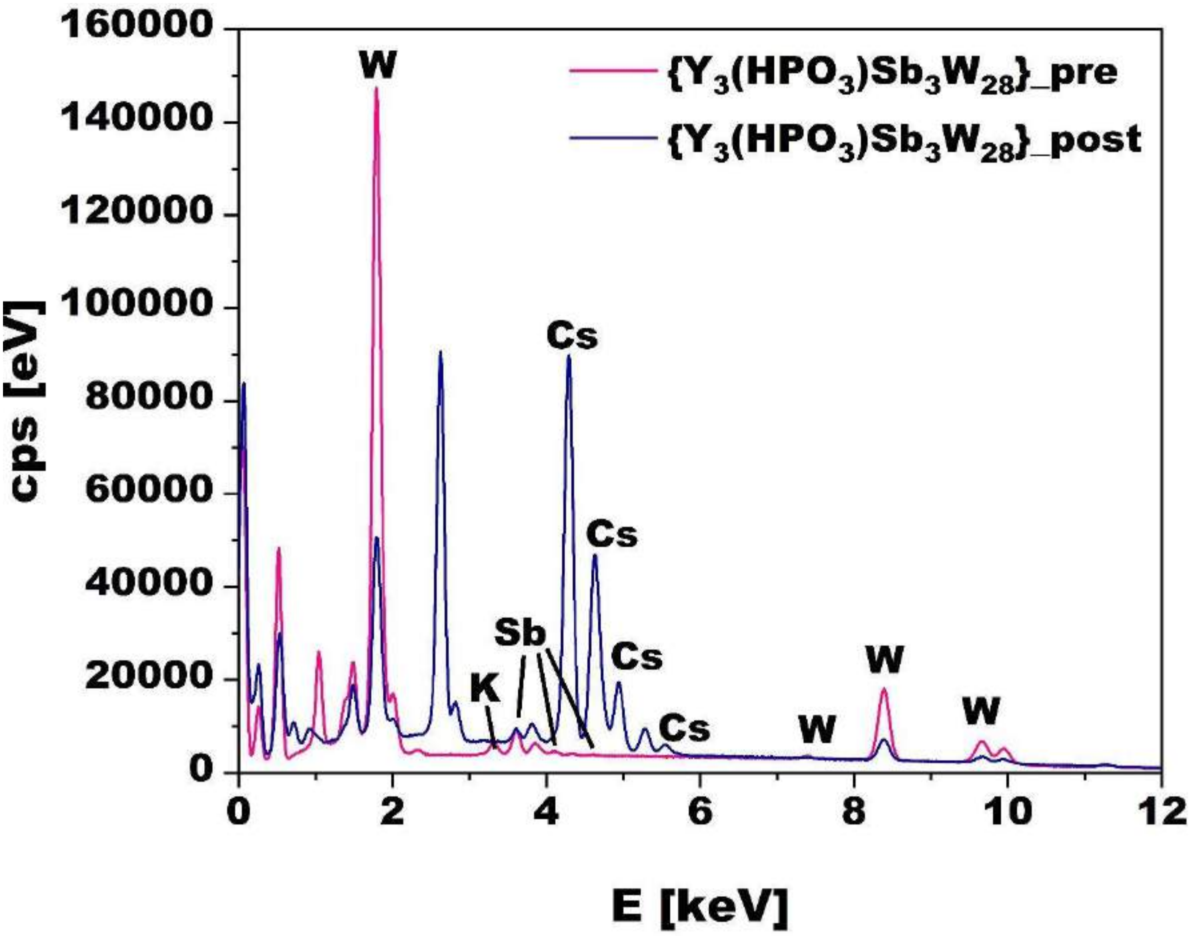

Figure S33. Energy dispersive X-ray analysis (EDX) spectrum of **{$Y_3(HPO_3)Sb_3W_{28}$}** (pre-catalysis) and **Cs{$Y_3(HPO_3)Sb_3W_{28}$}** (post-catalysis) (cps = counts per second). Element ratio found (calculated): Sb : W = 0.11 (0.11) (for **{$Y_3(HPO_3)Sb_3W_{28}$}**) and Sb : W = 0.08 (0.11) (for **Cs{$Y_3(HPO_3)Sb_3W_{28}$}**). Note that a decreased detected amount of Sb in the post-catalytically isolated cesium salt of the POM catalyst is attributed to overlaps with the Cs region.

8.3. Recyclability

The recyclability of **{$M_3(HPO_3)Sb_3W_{28}$}** and **{SbW_9}** as catalysts for the decontamination of DMNP was tested in a consecutive experiment by reloading the reaction mixture with DMNP substrate after ^{31}P-NMR measurements confirmed the DMNP conversion (> 98%) to the hydrolysis product DMP (**Fig. S34A**). A direct comparison of the turnover frequency (TOF) values obtained for **{$M_3(HPO_3)Sb_3W_{28}$}** and **{SbW_9}** in the first and the second reaction cycle indicates no significant change in the catalytic performance of the polyanions after one reaction cycle (**Fig. S35 – S38**, **Table S14**).

Following the confirmed end of the first reaction cycle as indicated by ^{31}P-NMR measurements (> 98% DMNP conversion, **Fig. S34 A**), a 200 µL stock solution of DMNP [12.6 mM] was added to the 600 µL NMR-sample containing 2.5 mM of the corresponding tungstoantimonate (**TA**)-catalyst **{$M_3(HPO_3)Sb_3W_{28}$}** or **{SbW_9}** leading to an 800 µL sample with the initial ratio **TA**:DMNP = 1:1.68. The reaction mixture was mixed thoroughly by flipping the NMR tube various times and monitored by ^{31}P-NMR spectroscopy. The ^{31}P-NMR integration values taken on reaction mixtures with < 50% substrate conversion (**Fig. S35 – S38**) were used to determine the amount of formed product. The TONs obtained were normalized by the number of three active metal centers in the case of **{$M_3(HPO_3)Sb_3W_{28}$}**. A comparison of the TOF values determined for the first and the second reaction cycle is given in **Table S14**.

Table S14. Comparison of turnover frequency (TOF) values of **{$M_3(HPO_3)Sb_3W_{28}$}** and **{SbW_9}** for the first and the second cycle of the hydrolytic conversion of DMNP to DMP.

Compound	1st cycle TOF [h^{-1}]	2nd cycle TOF [h^{-1}]
**{$Gd_3(HPO_3)Sb_3W_{28}$}**	0.021	0.023
**{$Y_3(HPO_3)Sb_3W_{28}$}**	0.017	0.016
**{$Yb_3(HPO_3)Sb_3W_{28}$}**	0.037	0.038
**{SbW_9}**	0.046	0.047

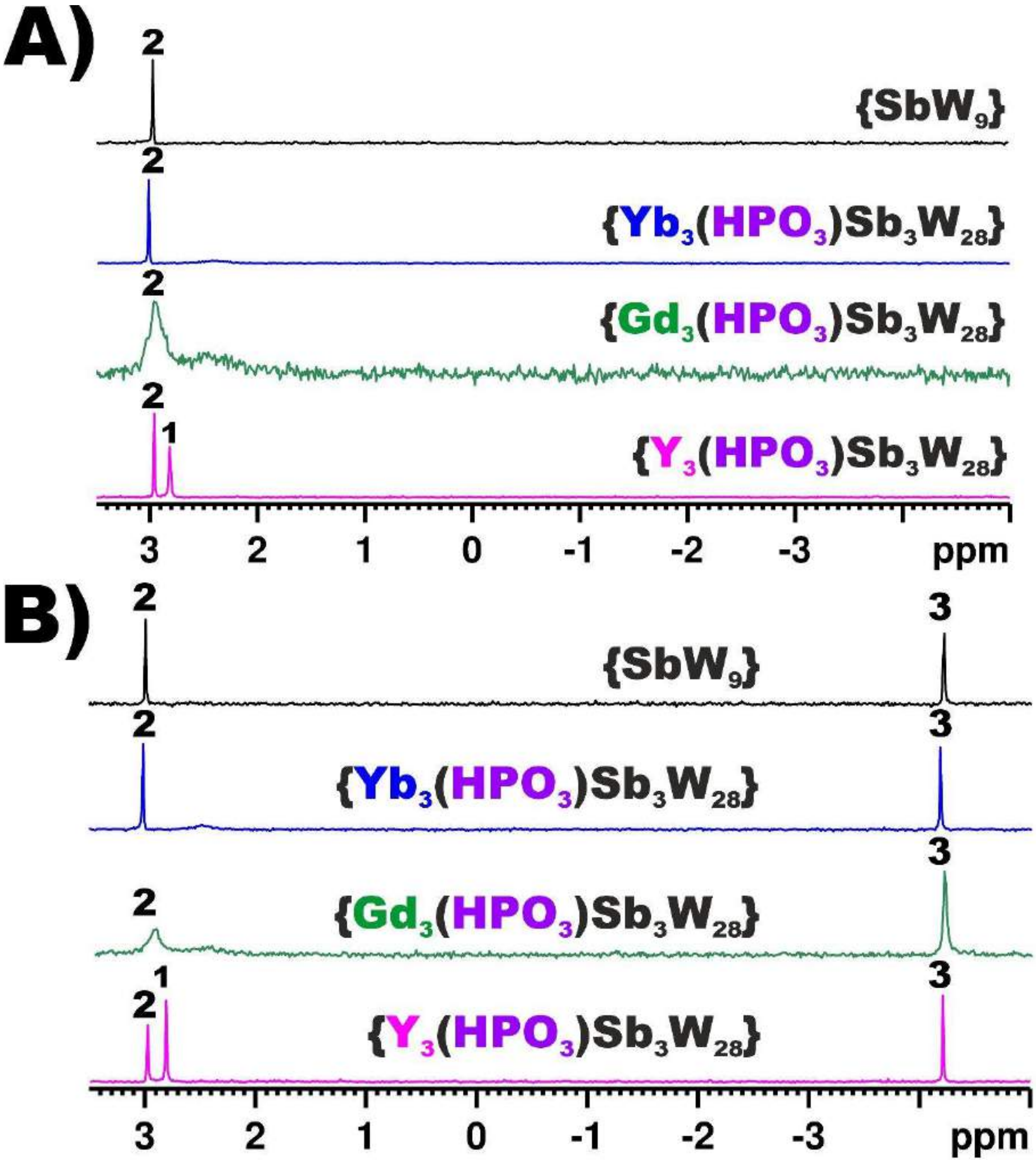

Figure S34. ^{31}P NMR spectra **A)** taken on catalytic reaction mixtures after seven days of incubation under optimized reaction conditions (2.5 mM **$\{Gd_3(HPO_3)Sb_3W_{28}\}$** (green), **$\{Y_3(HPO_3)Sb_3W_{28}\}$** (pink), **$\{Yb_3(HPO_3)Sb_3W_{28}\}$** (blue), **$\{SbW_9\}$** (black), 4.2 mM DMNP substrate in D_2O pD = 7 *via* NaOD at 25°C, or Tris-HCl [125 mM] for **$\{SbW_9\}$**, respectively) showing the complete hydrolysis of DMNP indicated by the singlet peak at 2.96 ppm corresponding to the hydrolysis product DMP (**2**). The virtually unchanged singlet peak at 2.81 ppm corresponds to the $\{HPO_3\}$ group (**1**) incorporated in the diamagnetic representative **$\{Y_3(HPO_3)Sb_3W_{28}\}$** suggesting solution stability of the isostructural series for up to seven days under the optimized turnover conditions. **B)** Subsequent re-loading of the solutions with new DMNP substrate results in the re-appearance of a singlet peak at 4.3 ppm which can be assigned to DMNP (**3**) thereby starting a second catalytic cycle.

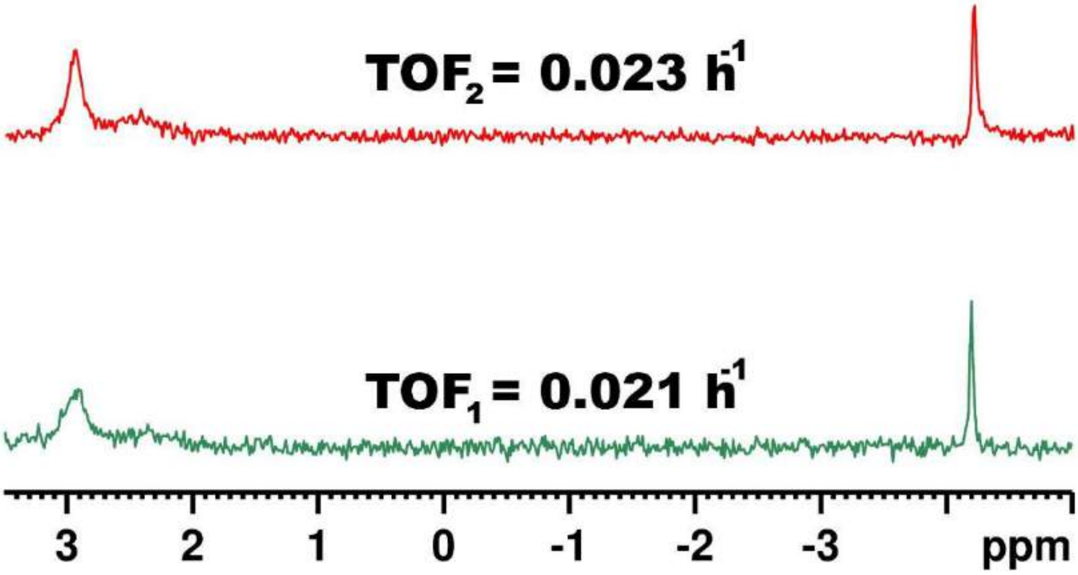

Figure S35. ^{31}P NMR spectra of a reaction mixture containing **{$Gd_3(HPO_3)Sb_3W_{28}$}** and the DMNP substrate in the ratio 1:1.68 in D_2O pD = 7 *via* NaOD at 25°C during the (green) and the second first reaction cycle (red), respectively.

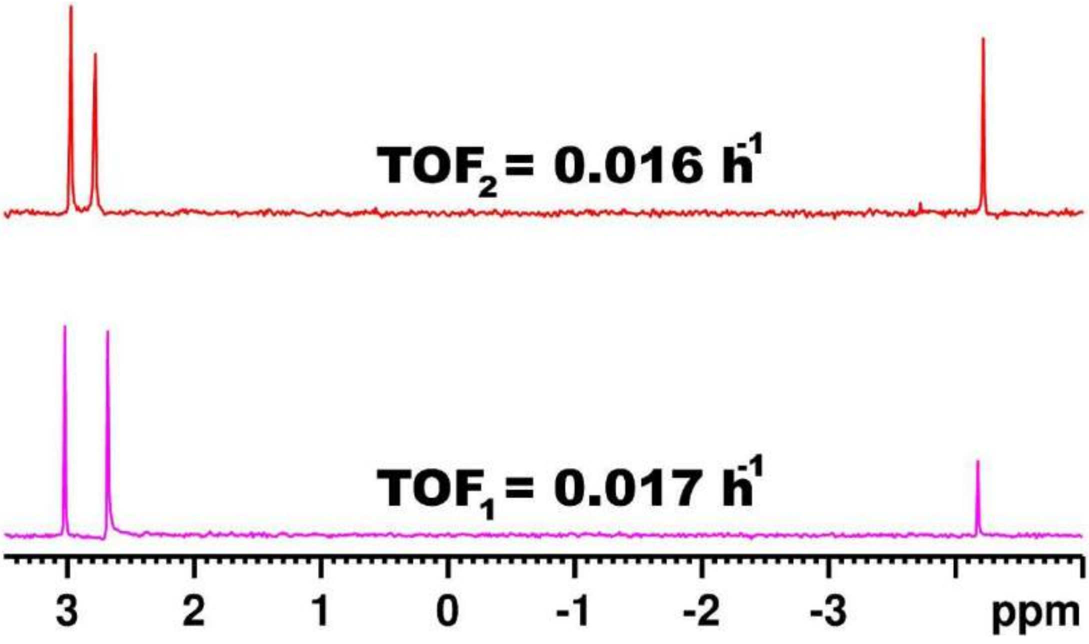

Figure S36. ^{31}P NMR spectra of a reaction mixture containing **{$Y_3(HPO_3)Sb_3W_{28}$}** and the DMNP substrate in the ratio 1:1.68 in D_2O pD = 7 *via* NaOD at 25°C during the (pink) and the second first reaction cycle (red), respectively.

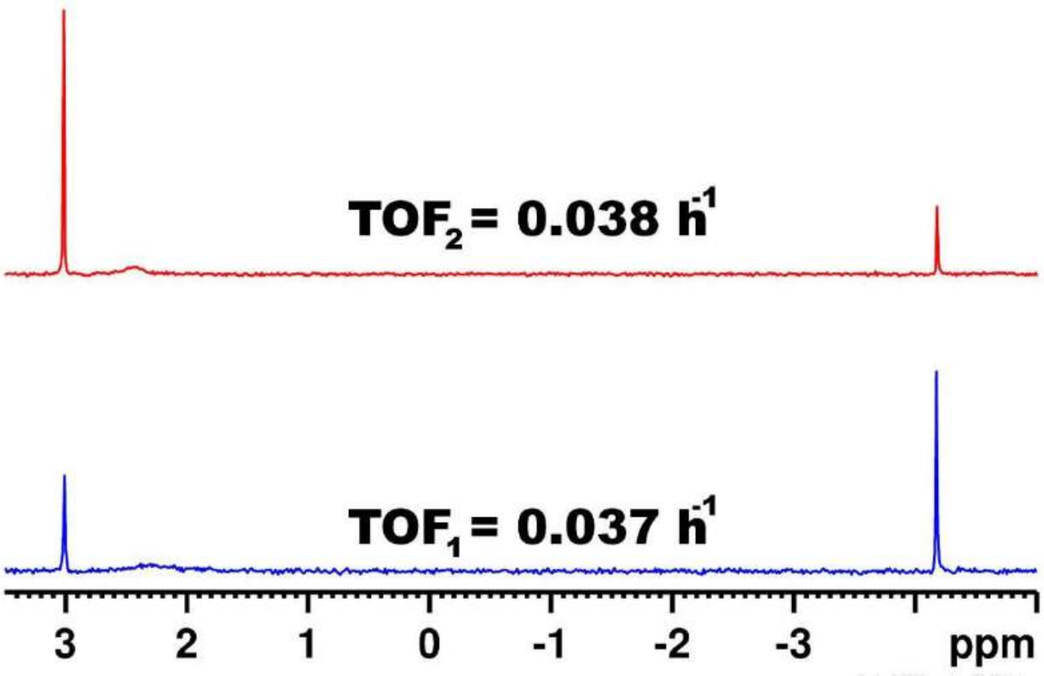

Figure S37. ^{31}P NMR spectra of a reaction mixture containing **{$Yb_3(HPO_3)Sb_3W_{28}$}** and the DMNP substrate in the ratio 1:1.68 in D_2O pD = 7 *via* NaOD at 25°C during the (blue) and the second first reaction cycle (red), respectively.

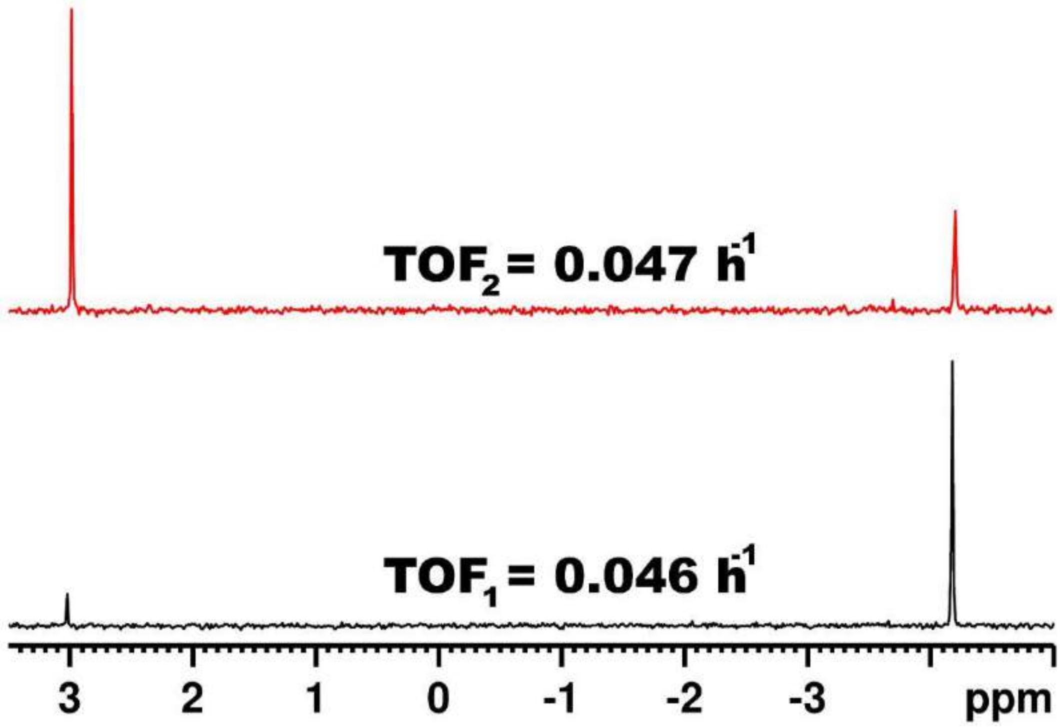

Figure S38. ^{31}P NMR spectra of a reaction mixture containing **{SbW_9}** and the DMNP substrate in the ratio 1:1.68 in Tris-HCl, 125 mM pD = 7 at 25°C during the (black) and the second first reaction cycle (red), respectively.

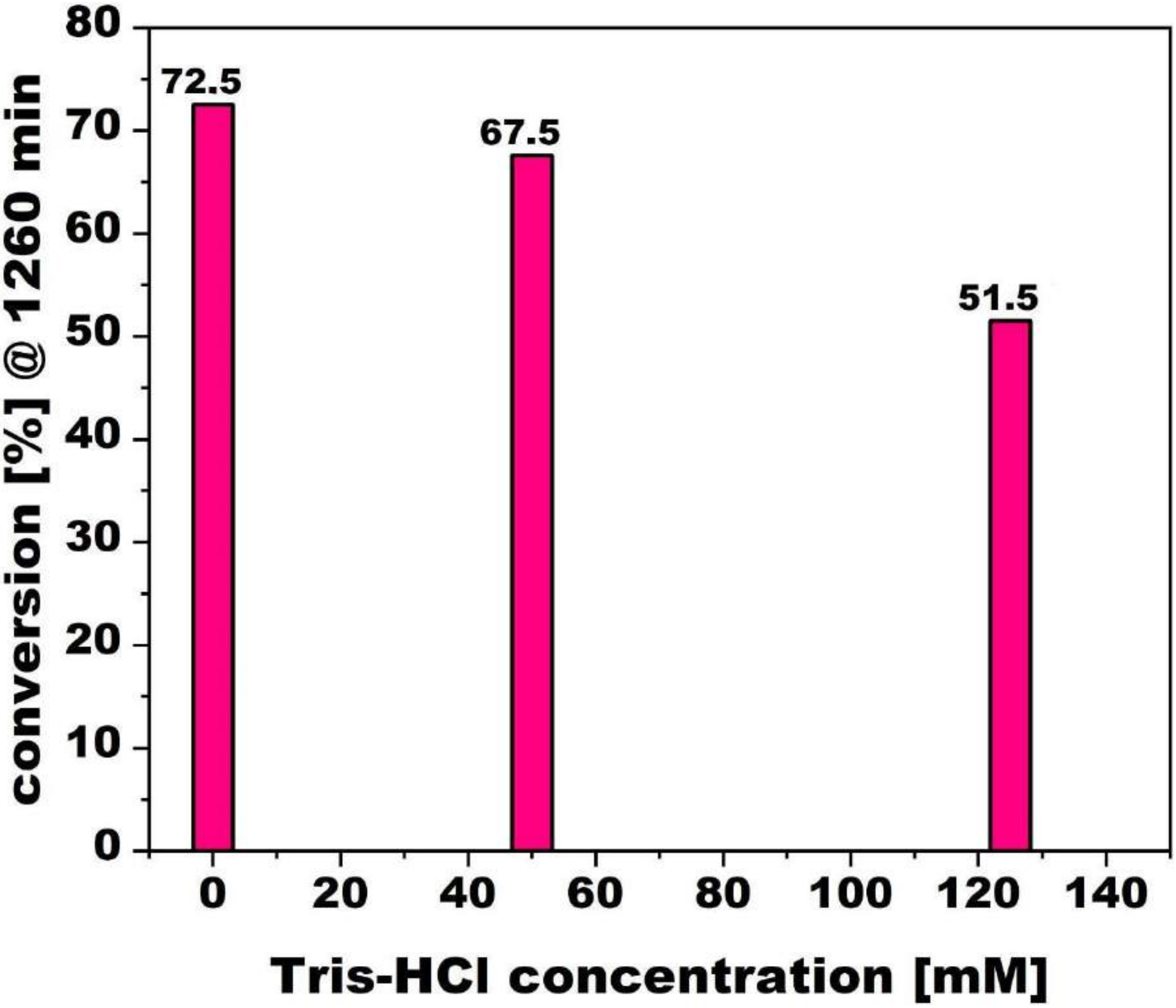

Figure S39. Comparison of the DMNP conversion by **{$Y_3(HPO_3)Sb_3W_{28}$}** after 1260 min of incubation upon increasing the Tris-HCl buffer concentration under elsewise identical reaction conditions (pD = 7.0, 25°C).

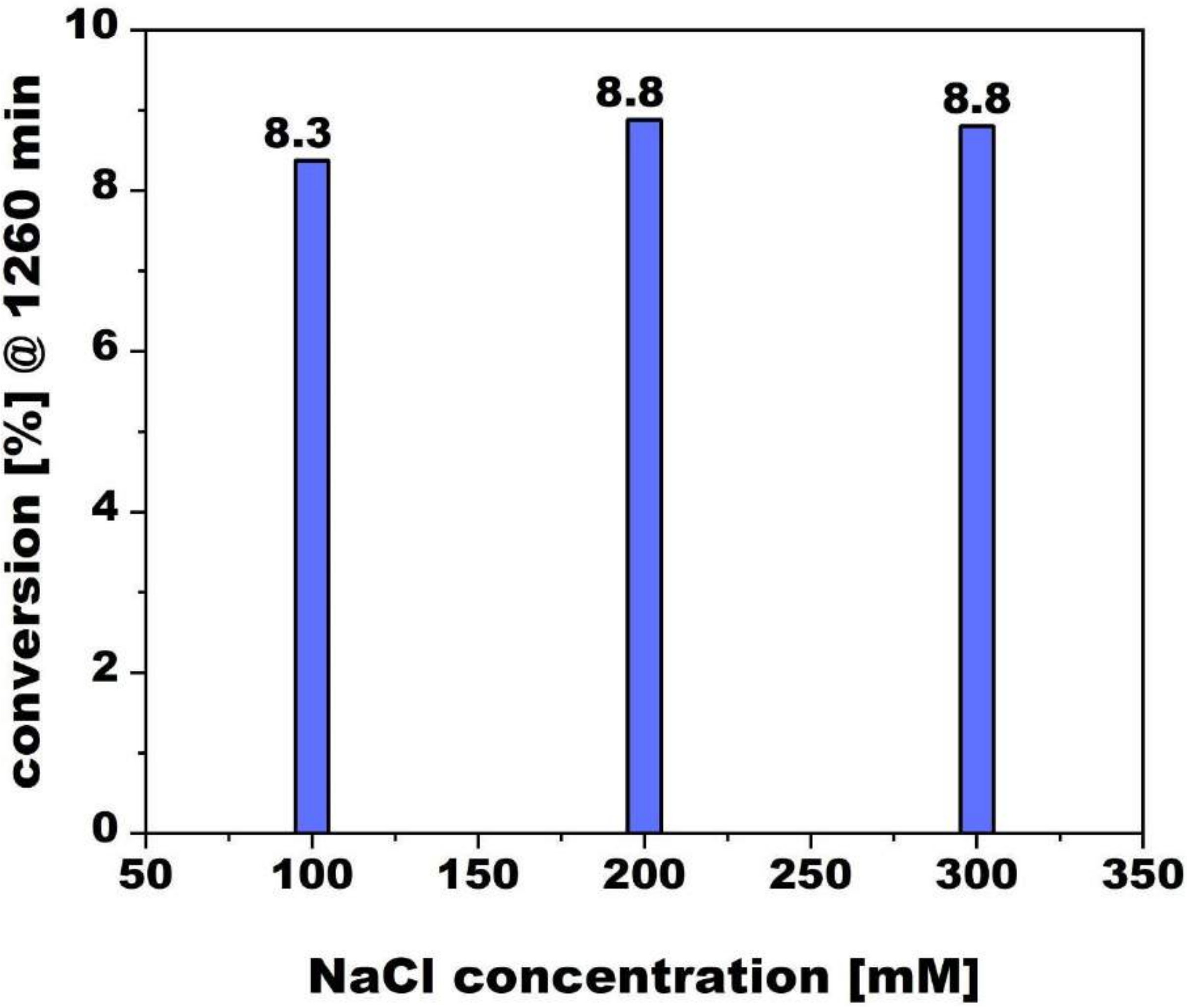

Figure S40. Comparison of the DMNP conversion by **{SbW$_9$}** after 1260 min of incubation upon increasing the NaCl concentration under elsewise identical reaction conditions (pD = 7.0 via NaOD, 25°C).

8.4. Control experiment employing a trilacunary lone-pair containing POT

To further explore the role of the basic terminal O-sites in the **{SbW$_9$}** for the observed catalytic activity, a control experiment employing $Na_9[B\text{-}\alpha\text{-}AsW_9O_{33}]\cdot 27H_2O$ **{AsW$_9$}**[2], which apart from an As^{III} primary hetero ion instead of Sb^{III}, is isostructural to **{SbW$_9$}** and features the same charge, was employed under elsewise identical catalytic conditions (Tris-HCl [125 mM], pD 7 at 25°C). Incubation of the reaction mixture for 24 h resulted in ~69.9% DMNP conversion corresponding to a calculated TOF value of 0.048 h^{-1} (**Figure S41**). This value is almost identical to the TOF calculated for **{SbW$_9$}** of 0.047 h^{-1} (**Table S14**) ultimately suggesting a general catalytic activity for trilacunary lone-pair containing POTs featuring accessible terminal O-sites in the presence of Tris.

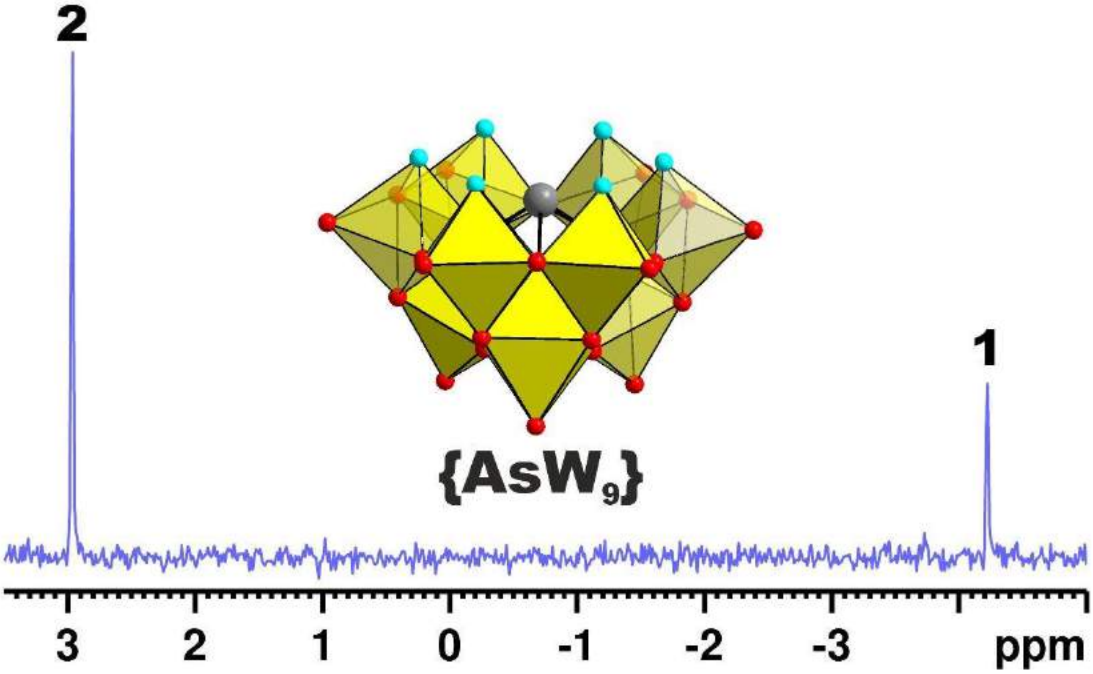

Figure S41. ^{31}P NMR spectrum showing the hydrolysis of DMNP (**1,** δ: -4.22 ppm) [4.2 mM] to nitrophenol (NP) and dimethyl phosphate (DMP) (**2**, δ: -2.96 ppm) after 24h incubation with **{AsW$_9$}** [2.5 mM] in Tris-HCl [125 mM] at pD 7.0 25 °C.

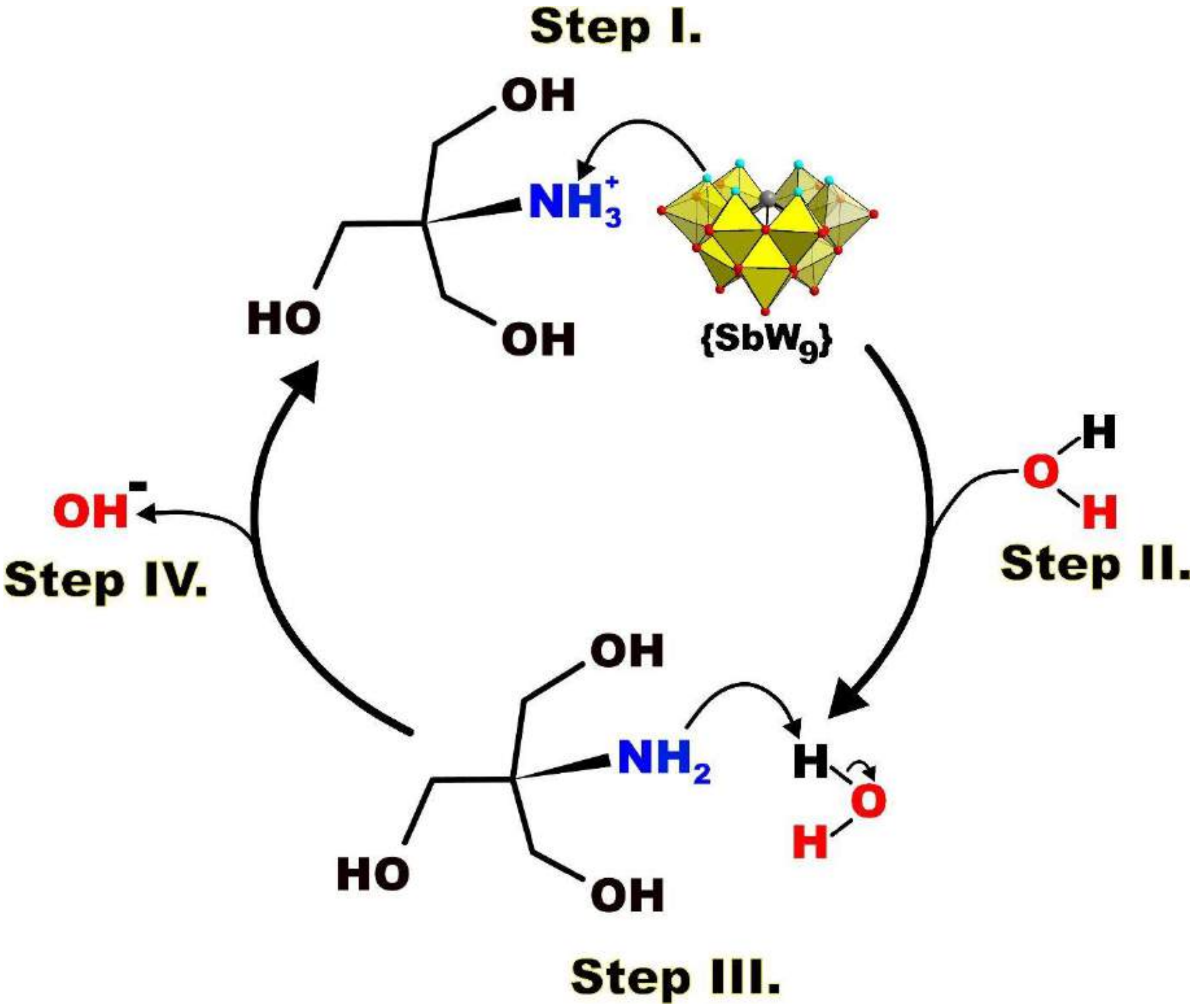

Scheme S2. Proposed mechanism of the Tris-mediated nucleophilic activation of H_2O. In a first step, the NH_3^+ amino group is deprotonated by the highly basic O-sites in the lacunary **{SbW$_9$}** POM (**I.**) followed by addition of H_2O (**II.**) which in turn is deprotonated by the Tris-base (generated in step I) acting as a proximal base (**III.**) resulting in the activation of H_2O by generating the OH^- - nucleophile (**IV.**). Color code: WO_6, yellow polyhedra; O, red balls; terminal O, turquoise balls; Sb, grey ball.

9. References

1 M. Bösing, I. Loose, H. Pohlmann and B. Krebs, *Chem. Eur. J.*, 1997, **3**, 1232–1237. DOI: 10.1002/chem.19970030810
2 A. P. Ginsberg, Ed., in *Inorganic Syntheses*, John Wiley & Sons, Inc., Hoboken, NJ, USA, 2007, pp. 1–25.
3 Bruker SAINT v7.68A Copyright © 2005-2016 Bruker AXS.
4 Sheldrick, G. M. SADABS University of Göttingen, Germany (1996).
5 Sheldrick, G. M. (1996) SHELXS. University of Göttingen, Germany.
6 G. M. Sheldrick, (1996) SHELXL. University of Göttingen, Germany.
7 O. V. Dolomanov, L. J. Bourhis, R. J. Gildea, J. A. K. Howard and H. Puschmann, *J. Appl. Crystallogr.*, 2009, **42**, 339–341. DOI: 10.1107/S0021889808042726
8 C. B. Hübschle, G. M. Sheldrick and B. Dittrich, *J. Appl. Crystallogr.*, 2011, **44**, 1281–1284. DOI: 10.1107/S0021889811043202
9 a) X. Xu, X. Liu, D. Wang, X. Liu, L. Chen and J. Zhao, *Inorg. Chem.*, 2021, **60**, 1037–1044. DOI: 10.1021/acs.inorgchem.0c03148; b) X. Xu, S. Xie, G. Yang and J. Zhao, *Inorg. Chem. Commun.*, 2020, **119**, 108073. DOI: 10.1016/j.inoche.2020.108073.
10 R. D. Shannon, *Acta Cryst, A*, 1976, **32**, 751–767. DOI: 10.1107/S0567739476001551
